# Small cell lung cancer: emerging subtypes, signaling pathways, and therapeutic vulnerabilities

**DOI:** 10.1186/s40164-024-00548-w

**Published:** 2024-08-05

**Authors:** Jing Zhang, Xiaoping Zeng, Qiji Guo, Zhenxin Sheng, Yan Chen, Shiyue Wan, Lele Zhang, Peng Zhang

**Affiliations:** Department of Thoracic Surgery, Shanghai Pulmonary Hospital, School of Medicine, Tongji University, Shanghai, 200433 China

**Keywords:** Small cell lung cancer, Emerging subtypes, Signaling pathways, Therapeutic vulnerabilities

## Abstract

Small cell lung cancer (SCLC) is a recalcitrant cancer characterized by early metastasis, rapid tumor growth and poor prognosis. In recent decades, the epidemiology, initiation and mutation characteristics of SCLC, as well as abnormal signaling pathways contributing to its progression, have been widely studied. Despite extensive investigation, fewer drugs have been approved for SCLC. Recent advancements in multi-omics studies have revealed diverse classifications of SCLC that are featured by distinct characteristics and therapeutic vulnerabilities. With the accumulation of SCLC samples, different subtypes of SCLC and specific treatments for these subtypes were further explored. The identification of different molecular subtypes has opened up novel avenues for the treatment of SCLC; however, the inconsistent and uncertain classification of SCLC has hindered the translation from basic research to clinical applications. Therefore, a comprehensives review is essential to conclude these emerging subtypes and related drugs targeting specific therapeutic vulnerabilities within abnormal signaling pathways. In this current review, we summarized the epidemiology, risk factors, mutation characteristics of and classification, related molecular pathways and treatments for SCLC. We hope that this review will facilitate the translation of molecular subtyping of SCLC from theory to clinical application.

## Introduction

Small cell lung cancer (SCLC) accounts for approximately 11% of all lung cancer cases [1]. Tobacco exposure is strongly associated with the incidence of SCLC. SCLC is considered as a recalcitrant tumor because of its early metastasis, rapid tumor growth and poor prognosis. Approximately 70% of patients have distant metastasis when they are first diagnosed with SCLC. Since the 1980s, a minority of very early-stage patients have received surgery as initial treatment and adjuvant platinum-based chemotherapy as follow-up treatment; most patients with advanced stage SCLC have received concurrent radiation and platinum-based chemotherapy [2]. Regardless of whether the patients had metastasis, the initial response rate to concurrent chemoradiotherapy was satisfactory. However, the response is short-lived, and the median survival time is less than one year [3]. Over the past decade, numerous clinical trials of combination immunotherapies have been conducted. Some of them have shown potential antitumor activity [4], and atezolizumab was recommended as a first-line treatment in 2019 [5]; however, the efficacy of immunotherapy still needs to be improved.

In the past few years, the prognosis of patients with non-small cell lung cancer (NSCLC) has improved significantly, mainly because of genotype-directed targeted therapies against tumor-specific somatic mutant forms [6]. In contrast, the identification of targetable driver mutations in SCLC lags behind that of other tumors, and SCLC has always been treated as a single disease entity. There have been no revolutionary new treatments for decades. SCLC has frequent copy number alterations (CNAs) and high tumor mutation burdens (TMBs). Genome sequencing and animal models have revealed more mutation hallmarks and related molecular pathways of SCLC. Some research groups have attempted to characterize the biologically distinct subtypes of SCLC based on the differential expression of transcriptional regulators. The molecular subtype classification is continuously being updated for the study of disease progression. A recognized subtype classification is urgently needed to develop more effective and personalized approaches for treatment.

In this review, we present the current trends regarding the epidemiology of, risk factors for, mutation characteristics of and treatments for SCLC. To explore the new directions of therapeutic research, we also highlight the latest discoveries in subtypes and related molecular pathways.

## Epidemiology

### Prevalence and prognosis

According to a recent population-based study, the annual number of new lung cancer cases worldwide is approximately 2.2 million, with SCLC accounting for 11% (232,000 cases) of these cases [1]. Previous research estimated that SCLC leads to 250,000 deaths worldwide each year [7]. The incidence of SCLC exhibits notable regional and sex disparities. In terms of regional prevalence trends, the incidence of SCLC exhibits a declining pattern in developed areas, while it exhibits a gradually increasing trend in underdeveloped areas [8]. In 2012, the highest incidence of SCLC among males was observed in Central and Eastern Europe as well as East Asia; over the same period, the highest incidence among females was observed in North America and Northern Europe [9]. A higher rate of smoking leads to a higher incidence of SCLC among males. This disparity is gradually diminishing toward equality, reflecting the declining rate of smoking among males and additional exposure to nonsmoking factors in females [10]. Variances in lung cancer incidence by country and sex are consistent with smoking trends, but there is a lag of 3–4 decades [9]. In recent decades, the implementation of smoking cessation initiatives has led to a gradual reduction in the relative SCLC incidence rate worldwide [11]. However, the disease burden caused by SCLC is expected to increase concomitantly with the increasing incidence of lung cancer in the future [12].

Owing to the rapid tumor growth and early metastasis of SCLC, the prognosis of SCLC is more unfavorable than that of other subtypes of lung cancer, with a median survival period of less than one year [3]. Thus, investigation of the orienting metastatic features of SCLC will be valuable. The most common sites of SCLC metastasis include brain, bone, adrenal glands, liver and contralateral lung. Recently, Chan et al. found that PLCG2 was significantly overexpressed in the metastatic sites. Among the metastatic sties, the liver and lymph node metastasis, the most common of metastatic site, had the highest level of PLCG2 [13]. Na et al*.* discovered that *KMT2C* was the frequently mutated gene in both primary tumor and metastatic samples. Subsequent in vivo and in vitro experiments further revealed that KMT2C-DNMT3A-MEIS/HOX axis was responsible for the metastasis in liver, lymph node, and other organs [14]. Collectively, these results offer a novel insight of SCLC metastasis and may be useful for the medical development.

### Risk factors

The occurrence of SCLC is closely linked to tobacco exposure both biologically and epidemiologically. Tobacco smoke contains more than 70 confirmed carcinogens, including more than 20 directly linked to the development of lung cancer [15]. The comprehensive genomic profiles of SCLC highlighted the significance of tobacco carcinogens in the initiation of SCLC [16]. The prevalence of ever-smokers among SCLC patients is as high as 94%, surpassing that of all subtypes of lung cancer [17]. Smoking is associated with a worse prognosis in patients diagnosed with SCLC [18, 19]. A significant dose‒response relationship has been observed between smoking intensity and the risk of developing SCLC, while cessation of smoking has been shown to be correlated with a reduction in SCLC incidence [20]. Moreover, secondhand smoke is a strong risk factor for SCLC. Individuals exposed to secondhand smoke are more susceptible to developing SCLC than nonsmokers [21]. Previous studies have shown that secondhand smoke is more strongly associated with SCLC than with other histological types of lung cancer [22].

**Fig. 1:**
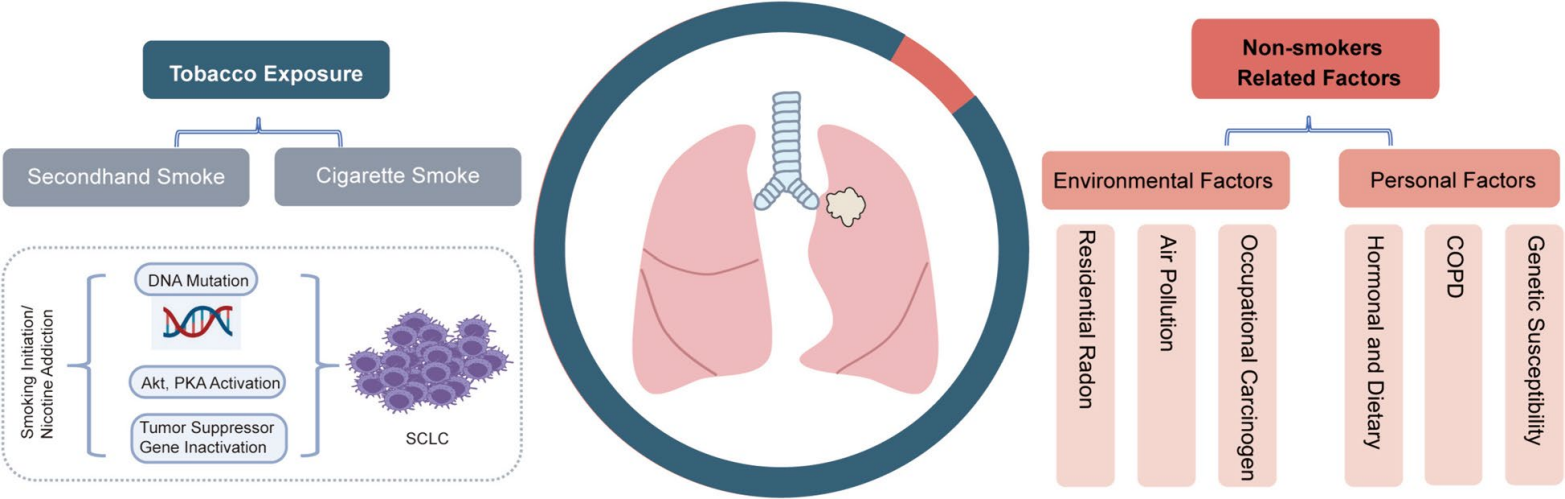
Risk factors associated with SCLC. The initiation of SCLC was associated with different factors, especially heavy smoking

The proportion of nonsmokers among SCLC patients ranges from approximately 2% to 2.5%, and a majority of them are female [23, 24]. In addition to tobacco exposure, risk factors for SCLC include residential radon, air pollution, occupational carcinogens, and hormonal and dietary factors. Among nonsmokers, residential radon is considered the leading cause of lung cancer [25]. Air pollutants that have been found to be closely associated with the incidence of SCLC mainly include nitrogen dioxide (NO_2_) and particulate matter 10 (PM_10_). Annual residential exposure to NO_2_ and PM_10_ is positively correlated with SCLC morbidity [26, 27]. Exposure to carcinogens involved in chemical industrial production processes, such as chloromethyl methyl ether, diesel engine exhaust, polycyclic aromatic hydrocarbons (PAHs), arsenic, silica asbestos, some heavy metals and their compounds, has been reported to contribute to lung cancer [28–30]. The intake of hormones and dietary habits also contribute to the prevention of lung cancer [31–34]. However, few studies have examined the effects of nonsmoking-related factors on SCLC alone, and the underlying mechanisms have yet to be fully elucidated. Furthermore, respiratory comorbidities such as COPD have been identified as independent risk factors for SCLC [20]. Genetic susceptibility also plays a significant role in the onset of SCLC; the details of this relationship will be further described in subsequent sections (Fig. 1).

## Origins of SCLC

**Fig. 2:**
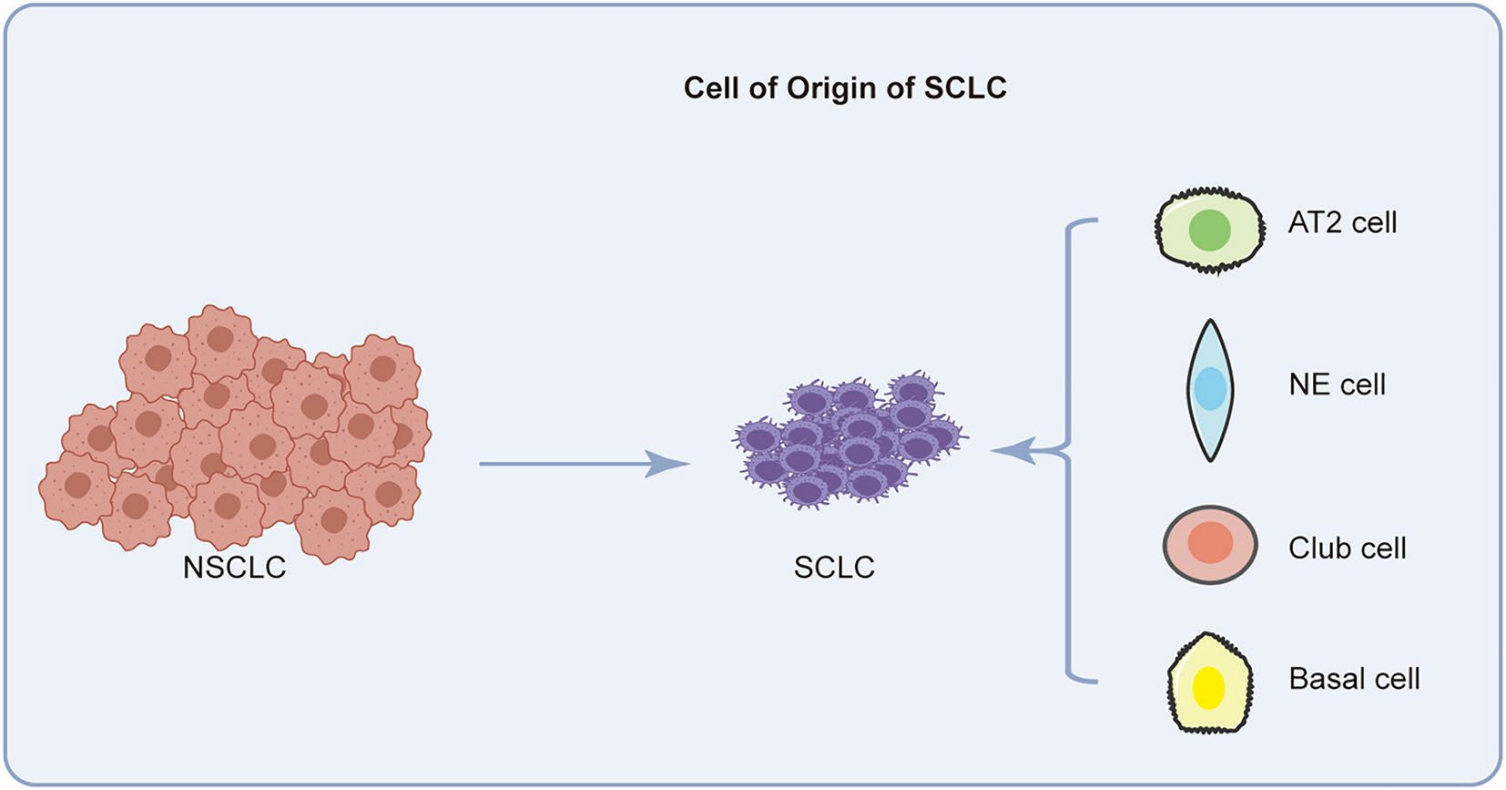
The cellular origin of SCLC. SCLC may originate from AT2 cells, NE cells, club cells and basal cells and transform from NSCLC cells

The rapid growth of SCLC often leads to advanced stage at the time of first diagnosis; therefore, few studies have exampled the cellular origins of SCLC [35]. Due to technological advancements in genome and transcriptome sequencing and the widespread access to cell-specific Cre recombinase and clustered regularly interspaced short palindromic repeats (CRISPR)-CRISPR-associated protein (Cas) 9 in SCLC cell lines and genetically engineered mouse models (GEMMs), insight into the origin of SCLC has gradually increased (Fig. 2).

Given the universal inactivation of *TP53* and *RB1* detected in humans, these genes are considered necessary for the tumorigenesis of SCLC [36, 37]. Initial mouse models revealed that the inactivation of *Rb1* and *Trp53* in lung epithelial cells via Adeno-Cre virus could lead to the neuroendocrine (NE) SCLC [38]. Subsequent triple-knockout GEMMs (*Rb1*/*Trp53*/*Pten*, *Rb1*/*Trp53*/*P130*) or *Rb1/Trp53* knockout GEMMs with *Myc, Nfib* or *Fgfr1* amplification could accelerate the initiation and progression of SCLC in lung epithelial cells [38–44]. Studies using the cell type-restricted Adeno-Cre virus in GEMMs with *Rb1* and *Trp53* inactivation have shown that NE cells and alveolar type 2 (AT2) cells expressing surfactant protein C (SPC) contribute to the formation of SCLC. In contrast to the study by Mollaoglu et al. [39], Chen et al. recently reported that AT2, club and NE cells can transform into SCLC cells via the combination of *Myc* amplification and the inactivation of *Rb1* and *Trp53* in a mouse model [44]. In the context of *Rb1*^*fl/fl*^*Trp53*^*fl/fl*^ GEMMs with *Fgfr1* amplification, SCLC can originate from tracheobronchial-basal cells expressing K14 [38–43]. Furthermore, the inhibition of *NOTCH*, *RB1* and *TP53* in human embryonic stem cells (hESCs) enables the generation of pulmonary NE cells (PNECs) and the formation of SCLC-like cells [45].

Distinct from the NE cell lineage, a variant form of SCLC with low NE features harbors markers of the tuft cell lineage and may originate from chemosensory epithelial cells of the primary and secondary bronchi [46]. Recent studies confirmed that non-NE SCLC cells could shift from the NE fate originating from PNECs via MYC/NOTCH or Yap/TAZ/NOTCH signaling [47, 48]. Additionally, some SCLCs can be derived from *EGFR*-, *ALK*-, ROS1- or *RET*-driven lung adenocarcinoma (LUAD) upon the acquisition of tyrosine kinase inhibitor (TKI) resistance [49–52]. Moreover, NSCLC patients with *EGFR/TP53/RB1* mutations or with apolipoprotein B mRNA editing enzyme catalytic polypeptide-like (APOBEC)–induced hypermutation may be at increased risk of transformation [53, 54]. Notably, transformation to SCLC may also occur in patients with NSCLC who receive immunotherapies [55–57]. For combined SCLC (CSCLC), including SCLC and NSCLC components, few studies have assessed its origin. Our previous study and research of Zhao et al. revealed that the same pluripotent clone with subsequent divergent oncogenic changes may be responsible for the different components of CSCLC [58, 59].

## Characterization of mutations in SCLC

Recalcitrant SCLC has been shown to exhibit extensive chromosomal aberrations and genomic alterations, which could contribute to the development and progression of SCLC with a high TMB. In a SCLC mouse model with *Trp53* and *Rb1* inactivation*,* DNA CNAs were detected on chromosomes 4, 8, 12, 14, 16 and 19 [60]. In human SCLC samples, amplification of chromosomes, including 1p, 1q, 3q, 5p, 8q, 14p, 14q, 18p and 18q, and deletions in 3p, 4p, 4q, 5q, 13q, 15q, 16q, 17p and 17q were detected in parallel to previous studies [16, 37]. Further multi-omics analysis revealed trans-associations on chromosome 5q, which are involved in DNA repair, replication and cell cycle progression [61].

Through comprehensive genomic analyses, SCLC was characterized by bi-allelic inactivation of *Tp53* and *Rb1* [16, 38]. Whole-genome sequencing (WGS) of 110 treatment-naive SCLC tumors revealed that the prevalence rates of genomic mutation in *TP53* and *RB1* were 94% and 78%, respectively [16]. Similarly, of the 3600 “real-world” SCLC patients, 92 and 74% had genomic mutations in *TP53* and *RB1*, respectively [62]. However, our multi-omics analysis revealed lower rates of *TP53* and *RB1* mutations in some SCLC patients (Table 1). The low rates of *TP53* and *RB1* mutations may be associated with different ancestry [62]. Moreover, other mechanisms may be responsible for *TP53* and *RB1* inactivation: (1) higher DNA deletions in *TP53* and *RB1* [61]; (2) chromothripsis, which is a catastrophic event that could lead to the inactivation of *RB1* [16]; (3) epigenetic alterations that may result in the inactivation of *TP53* and *RB1* [63, 64]; and (4) human viruses (such as human papillomaviruses, HPV) that can functionally inactivate *TP53* and *RB1* [62].

**Table 1:** Summary of publications with *TP53* and *RB1* mutations in SCLC

Publication	Year	Cases	*TP53* (%)	*RB1* (%)
Liu Q, et al. [61]	2024	112	72	56
George J et al. [271]	2024	65	100	100
Sivakumar S, et al. [62]	2023	3600	92	74
Wildey G, et al. [64]	2022	120	92	70
George J, et al. [16]	2015	110	94	78
Wagner AH, et al. [272]	2018	30	97	70
Rudin CM, et al. [37]	2012	30	73	55
Peifer M, et al. [36]	2012	27	93	67
Park S, et al. [273]	2019	166	92	70
Yokouchi H, et al. [274]	2020	79	54	NA
Udagawa H, et al. [275]	2018	204	74	42
Umemura S, et al. [276]	2014	51	80	39
Zhou H, et al. [277]	2021	40	88	72
Chen Y, et al. [89]	2021	19	42	32
Jiang L, et al. [75]	2016	99	82	62
**Range**			**42–100**	**32–100**

In addition to the *TP53* and *RB1* mutations, alterations in other genes, including *MYC*, *ZFHX3*, *PTEN*, and *NOTCH* family genes as well as *KMT2D, CREBBP* and *EP300,* were detected. MYC family genes, including *MYC*, *MYCL* and *MYCN*, are mutually exclusively expressed and exhibit approximately 20% amplification in SCLC [48]. In the lungs of GEMMs with inactivation of *Trp53* and *Rb1*, *MYC* amplification has been shown to promote the formation of SCLC, which is characterized by high aggression and metastasis and poor survival [39], thus leading to the dynamic evolution of SCLC [39, 48].

ZFHX3, a transcription factor (TF) with four homeodomains, 23 zinc finger domains and other motifs, has been suggested to be a tumor suppressor for different cancers [65]. In metastatic tumors, frequent mutations in *ZFHX3* can be detected [66, 67]. In our recent study, we found that the rate of *ZFHX3* mutation was 19% in Chinese patients, which is higher than that in a previous study, and that *ZFHX3* mutation could serve as a biomarker for immunotherapy response in SCLC patients [61].

*NOTCH* family genes were recurrently mutated with a pattern of frequent inactivation. Mutation of the *NOTCH* signaling pathway occurs in approximately 25% of human SCLC cases [16]. Activation of the NOTCH signaling pathway inhibited tumor growth and increased survival. Moreover, the active NOTCH signaling pathway can increase the expression of antigen processing and presentation machinery (APM) genes in SCLC [68]. *PTEN* is an oncogenic phosphatidylinositol 3 kinase (PI3K) inhibitor that is lost in SCLC. A significant reduction in tumor latency and overall survival (OS) can be observed in *Trp53/Rb1/PTEN* triple-knockout (TKO) GEMMs [60]. Approximately 8% of SCLC have *KMT2D* mutations, including truncating nonsense/frameshift/splice site mutations [69]. *KMT2D* deletion has been reported to lead to significant defects in cell type-specific gene expression and cell differentiation [70]. Additionally, deletions and truncating mutations of *CREBBP* and *EP300* in the histone acetyltransferase (HAT) domain are frequently found in SCLC [71].

## Evolutionary dynamics of SCLC subtypes

From a clinical perspective, SCLC is considered as a single disease entity, which may explain the failure of different drugs that have been examined for its treatment. By integrating data on human tumors, cell lines and different mouse models, distinct subtypes of SCLC have been characterized, and precise subtype-specific treatments have been proposed [72]. In the current section, investigations of SCLC classification will be reviewed.

Histologically, SCLC is first dichotomized into classic and variant subtypes on the basis of morphology and growth characteristics [73]. Further investigation has revealed the heterogeneity of SCLC based on NE features, which is similar to the above findings. The low-NE subtype has morphological features of the variant subgroup and grows in a loose aggregated or single form, unlike the high-NE subtype [74].

From a genomic perspective, comprehensive whole-exome or whole-genome sequencing of SCLC has demonstrated only universal mutations in *TP53* and *RB1*. Moreover, unlike LUAD, no genetic subtypes of SCLC and no revolutionized specific therapeutic vulnerabilities have been identified [16, 36, 37, 75]. With the accumulation of human SCLC tumors, a “real-world” study identified three potential genetic subtypes: a cohort without *TP53*/*RB1* alteration, a cohort with *STK11* mutation, and a cohort that may transform from NSCLC with typical oncogenic driver mutations [62].

**Fig. 3:**
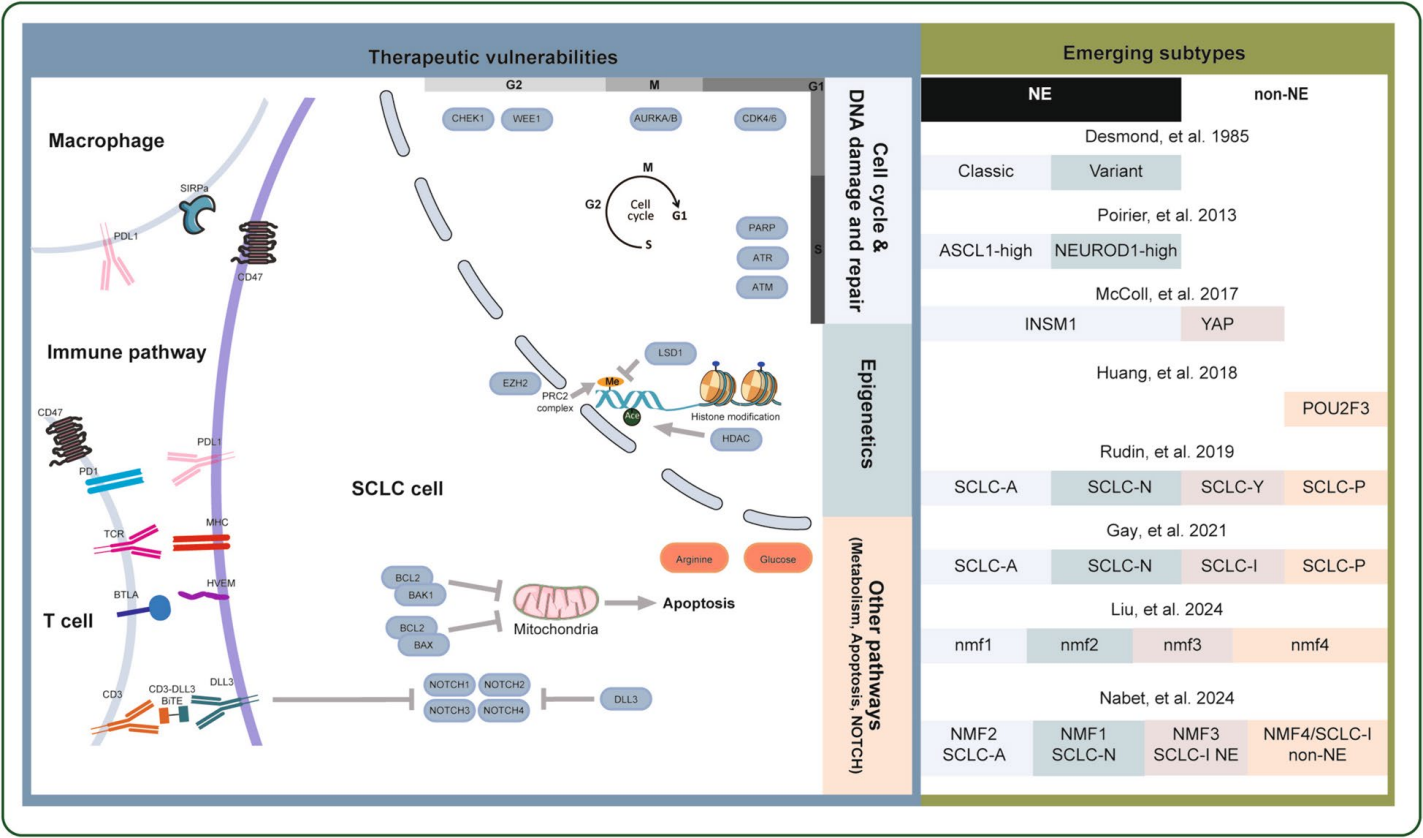
Therapeutic vulnerabilities and emerging subtypes of SCLC. A schematic summarizing the proposed potential therapeutic targets and emerging molecular subtypes of SCLC is shown. On the left side of the diagram, potential therapeutic targets, including those involved in the cell cycle and DNA damage and repair pathway, epigenetics pathway, metabolism pathway, NOTCH pathway, apoptosis pathway and tumor immunity, are displayed. (The activation effect is denoted by an arrow, while the inhibition effect is represented by a vertical bar). On the right side, the evolution of SCLC subtypes is shown in chronological order [46, 61, 72, 73, 76, 78, 80, 82]. Abbreviations are shown below. Achaete-scute homolog 1, ASCL1; neurogenic differentiation factor 1, NEUROD1; POU class 2 homeobox 3, POU2F3; yes-associated protein 1, YAP1; insulinoma-associated protein 1, INSM1; neuroendocrine, NE

Given the integrated analysis of human and mouse model data, unexpected molecular classification of SCLC has been proposed. The initial exploration of the selective susceptibility of Seneca Valley virus (SVV-001) to various subtypes of SCLC revealed that two TFs, Achaete-scute homolog 1 (ASCL1) and neurogenic differentiation factor 1 (NEUROD1), play key roles in NE differentiation [76]. Subsequent in vivo and in vitro experiments further confirmed that ASCL1 and NEUROD1 drive SCLC subtypes [39, 48, 77]. Unsupervised clustering analysis of a large panel of SCLC cell lines revealed that insulinoma-associated protein 1 (INSM1), an NE TF, and yes-associated protein 1 (YAP1), a vital mediator activated in the Hippo signaling pathway, may define two subtypes of SCLC [78]. Subtypes with high YAP1 expression displayed low levels of ASCL1 and NEUROD1. Conversely, variable levels of ASCL1 and NEUROD1 can be detected in subtypes with higher INSM1 expression [78]. CRISPR screening of SCLC cell lines revealed that POU class 2 homeobox 3 (POU2F3), a powerful TF, was expressed exclusively and necessary in the variant subtype of SCLC lacking NE marker expression [46]. Thereafter, synthesized analysis of both SCLC cell lines and tumor RNA data further suggested that SCLC can be definitively distinguished by the TFs ASCL1, NEUROD1, YAP1 and POU2F3 [72]. The activation of NOTCH signaling pathways by MYC in ASCL1 subtypes can drive the activation of the NEUROD1 and YAP1 subtypes in an orderly manner, indicating that no distinct subtypes instead of different stages of dynamic evolution may exist in SCLC [48]. When exploring the expression of the four TFs at the protein level, their expression profiles tended to be more heterogeneous [79]. POU2F3, which was uniquely expressed in 7% of SCLC patients, showed mutually exclusive associations with ASCL1 and NEUROD1. However, low levels of YAP1 coexisted with other subtypes. For the NE subtype of SCLC, the coexpression ratio of ASCL1 and NEUROD1 was more prevalent than that of ASCL1-positive or NEUROD1-positive SCLC [79]. To better refine the subtypes of SCLC, RNA sequencing data from surgically resected SCLC (n = 81) and the IMpower 133 clinical trial (n = 276) revealed four SCLC subtypes: the ASCL1, NEUROD1, POU2F3 and Inflamed subtypes. Notably, the Inflamed subtype had low levels of the three TFs and can strikingly benefit from immune checkpoint inhibitors (ICIs) [80]. A subsequent study demonstrated that these subtypes can be identified by tumor- and circulation-free DNA methylation [81]. In contrast to the above classification, four different subtypes were discerned by de novo non-negative matrix factorization (NMF) using the IMpower 133 data [82]. Robustly high levels of NEUROD1 and ASCL1 can be detected in the NMF1 and NMF2 subtypes, respectively. However, inflamed features can be found in both NMF3 and NMF4. In contrast to NMF3, NMF4 had unique POU2F3 expression and non-NE features. In addition, patients with the NMF3 subtypes with NE features and low levels of T-effector-high/tumor-associated macrophages (TAMs) can benefit from immunotherapy [82] (Fig. 3).

Emerging evidence substantiates that multi-omics analysis of tumors can offer a better understanding of disease and may provide a more specific therapeutic regimen [83–85]. Recently, our team has demonstrated four novel subtypes of SCLC by synthesizing multi-omics data, including mRNA, protein and phosphorylation data [61]. The nmf1 subtype is characterized by cell cycle progression and DNA damage, suggesting that this subtype has a high proliferation rate. The nmf2 subtype, which has a lower amount of multi-omics data, exhibits the highest TMB and highest expression of delta-like ligand-3 (delta-like ligand 3, DLL3). The nmf3 subtype, which is enriched in the extracellular matrix and focal adhesion pathway at the protein level, exhibits robust receptor tyrosine kinase (RTK) signaling pathway activity according to the phosphorylation data. Uniquely, the nmf4 subtype is mainly associated with RNA metabolism pathways and a high number of MYC targets.

To explore the association between multi-omics subtypes and previous subtypes, the TU-SCLC cohort was grouped into four subtypes using the NMF-defined gene list reported by Gay et al. [80]. The correlation between multi-omics and the established subtypes in the TU cohort was significant (P = 1.71E-20, Fisher’s exact test). Additionally, the nmf1 subtypes with half ASCL1-driven and half NEUROD1-driven tumors had higher levels of ASCL1 and NEUROD1; the nmf2 subtype had the highest number of ASCL1-driven tumors coupled with higher levels of DLL3; and the nmf3 subtype with a non-NE phenotype had mostly inflamed tumors. The nmf4 subtype with lower ASCL1 and NEUROD1 expression included all POU2F3-driven tumors except for one with high YAP1 expression.

Apart from the above subtypes of SCLC, other classifications have also been proposed, which may be useful for identifying potential distinct therapeutic vulnerabilities. Relying on the DNA methylation and RNA-seq data of primary SCLC, three distinct subtypes (M1, M2 and SQ-P for the methylation cluster and E1, E2 and SQ-P for the gene expression cluster) were identified by Poirier et al. [86]. High levels of NEUROD1 and low levels of ASCL1 were observed in the E1 cluster. In contrast, the opposite results were detected in the E2 cluster. For the SQ-P cluster, similar gene expression profiles were discerned compared with those of lung squamous cell carcinomas, which lacked NE markers. Intriguingly, unsupervised hierarchical clustering of RNA-seq data revealed two groups [16]. Group 2 represented the majority of SCLC patients and exhibited high levels of CHGA, GRP, ASCL1 and DLK1. Furthermore, Wooten et al. defined four subtypes of SCLC via systems-level analyses of RNA-seq data from SCLC cell lines, human tumors and patient-derived tumor xenograft (PDX)/cell line-derived xenograft (CDX) mice [87]. Under these conditions, a canonical NE subtype referred to as the ASCL1 group, an ASCL1 + NE variant assigned as the NEv2 group, or SCLC-A2, an NE variant subtype termed the NEv1 group aligning with the NEUROD1 group, and a non-NE variant subtype termed the YAP1 group, were identified. Unbiased hierarchical clustering of SCLC CDX RNA-seq data revealed four subtypes, namely, ASCL1, NEUROD1, POU2F3 and the TF ATOH1, which are important for neuronal differentiation [88]. Recently, another four clusters of SCLC were classified by Chen et al. [89]. Cluster 1 characterized by low levels of ASCL1 and NEUROD1 and high levels of POU2F3 and NOTCH2, was associated with immune-related features termed the immune subtype. Clusters 2 and 3 were parallel to the ASCL1 and NEUROD1 groups, respectively. Cluster 4, which was characterized by the expression of Clara cell secretory protein (CCSP), may originate from club cells and was therefore defined as the SCLC-C group.

Different from bulk RNA sequencing used for classification, single-cell RNA sequencing (scRNA-seq) can better address the heterogeneity of SCLC. In contrast to LUAD, the inter-patient and intra-tumor heterogeneity of SCLC malignant cells was much higher. Based on the distinct expression patterns of canonical TFs, Chan et al. discovered the most likely classifications of each cell, and identified the major subclone of each tissue as ASCL1, NEUROD1, POU2F3, except YAP1 [13]. Similarly, our study concerning the analysis of our scRNA-seq in metastatic SCLC also identified the different ASCL1 and NEUROD1 expression patterns, which further uncovered the diversity of inter-patient and intra-tumor heterogeneity [90].

Taken together, similarities have been detected among these classifications of SCLC. However, uniform and rigorous consensus clustering of SCLC patients, which may overcome the lack of therapeutic vulnerabilities, is still under debate, and further analysis is needed.

## Precise therapeutic vulnerabilities of SCLC subtypes

**Fig. 4:**
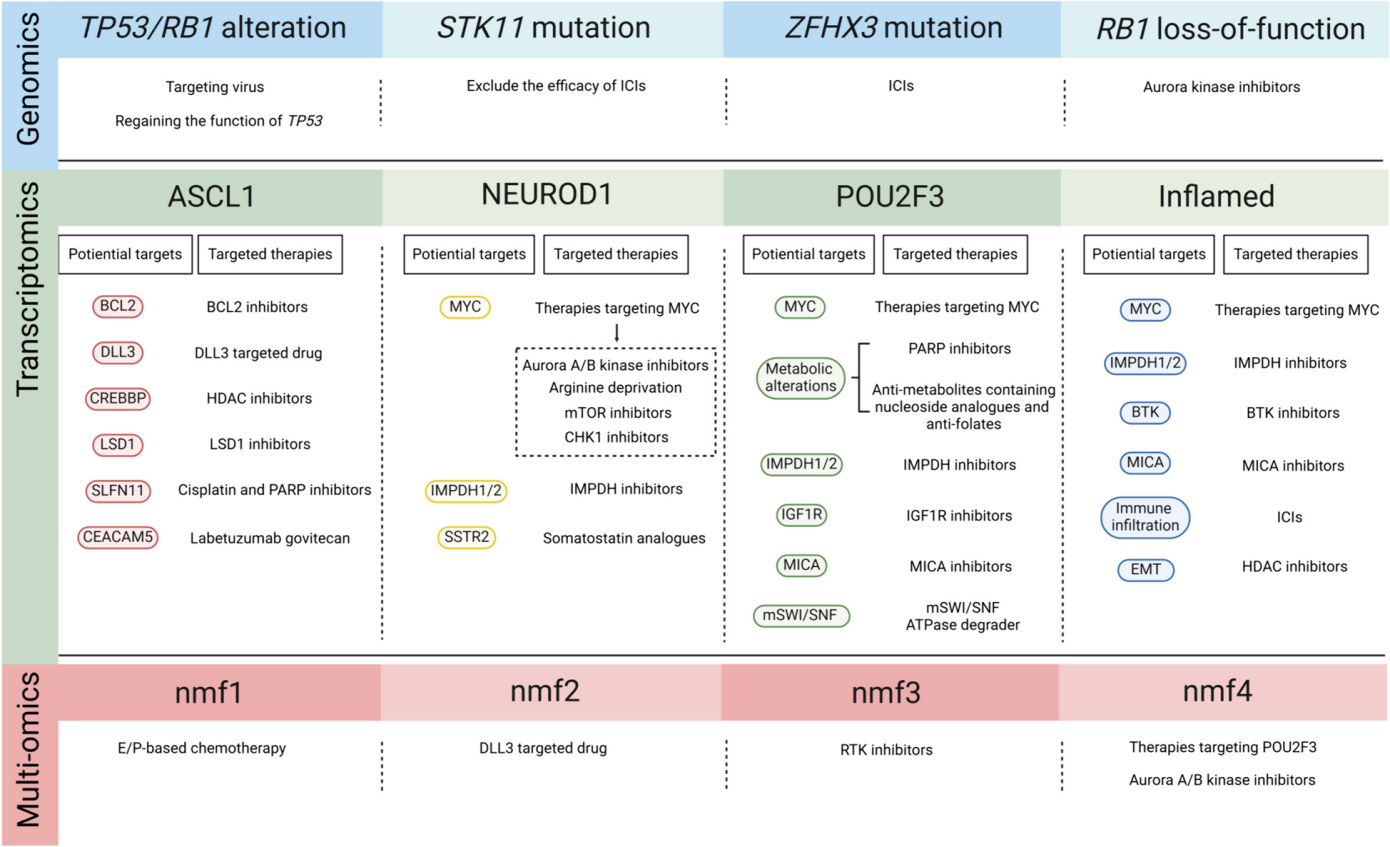
Therapeutic vulnerabilities of specific SCLC clusters. The SCLC clusters from the perspectives of genomics, transcriptomics and multi-omics are shown, and their potential available therapeutic vulnerabilities are discussed

Subtype-specific molecular characterization and alterations in key signaling pathways provide a basis for exploring specific therapeutic strategies (Fig. 4). Three potential genetic subtypes identified in the “real-world” study showed that the cohort without *TP53*/*RB1* alteration may benefit from targeting the virus or regaining the function of *TP53*; the cohort with *STK11* mutation may exclude the efficacy of ICIs, and the cohort transformed from NSCLC with typical oncogenic driver mutations may avoid transformation with better treatment [62]. Apart from the above genetic subtypes, our recent study revealed that patients with the *ZFHX3* mutation subtype of SCLC may benefit from ICIs [61]. Unexpectedly, Aurora kinase A (AURKA) inhibitors have been shown to have a durable effect on SCLC with *RB1* loss of function [91].

The heterogeneity of SCLC subtypes driven by TFs has strengthened the exploration of precise therapeutic options. Chromatin immunoprecipitation sequencing (ChIP-seq) revealed the unique downstream targets of ASCL1 and NEUROD1, providing the initial insights into the specific treatment involved [77]. The functional oncogenes targeted by ASCL1 in SCLC include BCL2, NFIB, SOX2, RET, MYCL1, and DLL3. In vivo and in vitro experiments confirmed that a BCL2 inhibitor can induce the apoptosis of SCLC cell lines with high BCL2 levels, indicating that a BCL2 inhibitor may be effective for treating the ASCL1 subtype of SCLC [92]. Similarly, DLL3, an inhibitory ligand of the NOTCH pathway, may be a prospective target for the ASCL1 subtype, despite failure of the DLL3-targeted drug (rovalpituzumab tesirine, Rova-T) in SCLC, which may be closely associated with the undistinguished population [93, 94]. Additionally, *CREBBP*, which encodes a histone acetyltransferase, was found to have frequent genetic mutations in SCLC [16, 61]. The loss of *CREBBP* in the *Rb1/Trp53* GEMM (mainly belonging to the ASCL1 subtype) drastically contributed to the progression of SCLC, which can show an exceptional response to the histone deacetylase (HDAC) inhibitor pracinostat [71]. Suppression of the histone demethylase lysine-specific demethylase 1 (LSD1) with the selective inhibitor ORY-1001 can reactivate the NOTCH pathway and attenuate the level of ASCL1, suggesting the potential therapeutic potential of LSD inhibitors in the ASCL1 subtype [95]. Within the ASCL1 subtype, a bimodal distribution of Schlafen family member 11 (SLFN11) was detected [95]. The critical response to cisplatin and the poly (ADP-ribose) polymerase (PARP) inhibitor olaparib can be confirmed in the ASCL1 subtype, which has a high level of SLFN11 [77, 80]. Carcinoembryonic antigen-related cell adhesion molecule 5 (CEACAM5), another highly expressed target gene in the ASCL1 subtype of SCLC, can be targeted by labetuzumab govitecan [80].

The level of MYC, a transcriptional target of NEUROD1, was amplified or overexpressed in ASCL1-low SCLC subtypes and correlated with poor prognosis and treatment resistance [39]. A targeted drug screening experiment demonstrated that SCLC patients with high MYC levels were more vulnerable to treatment with an Aurora kinase A/B (AURKA/AURKB) inhibitor, which indicated that patients with the NEUROD1 subtype may benefit from Aurora kinase inhibitors [39, 96]. In addition, inosine monophosphate dehydrogenase-1 and -2 (IMPDH1 and IMPDH2), which are selectively expressed in ASCL1-low SCLC, are downstream targets of MYC. The use of an IMPDH inhibitor can better suppress SCLC growth [97]. Similarly, MYC-driven SCLC is vulnerable to arginine deprivation and mTOR inhibition in combination with a checkpoint kinase 1 (CHK1) inhibitor [98, 99]. Taken together, these findings indicate that these targeted inhibitors may be potential therapeutic candidates for treating NEUROD1 and other ASCL1-low subtypes in which MYC is overexpressed. High levels of somatostatin receptor 2 (SSTR2), which is a well-established target of somatostatin analogs, were detected in the NEUROD1 subtype.

The POU2F3 and Inflamed subtypes, which are rare subsets of SCLC, have unique therapeutic vulnerabilities. CRISPR screening revealed the essential role of activating the insulin-like growth factor 1 receptor (IGF1R) signaling pathway in the POU2F3 subtype. The IGF1R inhibitor linsitinib may be a promising targeted drug for the POU2F3 subtype. A recent study revealed that compared with other subtypes, the POU2F3 subtype is more vulnerable to PARP inhibitors, antimetabolites containing nucleoside analogs, antifolates and mammalian switch/sucrose non-fermentable (mSWI/SNF) ATPase degrader [80, 100]. *MICA,* which encodes MCH class I polypeptide–related sequence A, is highly expressed in the POU2F3 and Inflamed subtypes and can be targeted by IPH43 in these two subtypes [80]. The Inflamed subtype is a novel group that has the highest immune infiltration, cytolytic activity score, highest expression of Bruton’s tyrosine kinase (BTK) and mesenchymal status in SCLC. The available evidence has shown that the Inflamed subtype may benefit from ICIs, BTK inhibitors, and HDAC inhibitors, which can restore epithelial markers [80].

In terms of the unique therapeutic vulnerabilities of multi-omics, external data were integrated with functional experiments. The nmf1 subtype associated with exceptionally high NE scores, a high cell proliferation rate, high E2F activity and replication stress may be better targeted by drugs that exacerbate genome instability. Thus, we hypothesized that E/P-based chemotherapy may be the proper treatment option for these nmf1-subtype tumors [61]. Emerging evidence has shown that concurrent inhibition of Ataxia telangiectasia and Rad3-related protein (ATR) and DNA topoisomerase I (TOP1) can result in durable tumor regression in SCLC characterized by high replication stress [101]. In contrast, DLL3-targeted drugs may be the best choice for the nmf2 subtype with the highest DLL3 level, which was confirmed in the George et al. dataset [16, 61]. The nmf3 subtype, characterized by elevated RTK signaling pathway activity, was shown to benefit from RTK inhibitors in PDX models. In contrast to the findings of a previous study, the nmf4 subtype was confirmed to have the highest MYC and POU2F3 levels via multi-omics and immunohistochemical assays. Subsequent experiments demonstrated that the nmf4 subtype may be suitable for AURKA/B inhibitors.

In conclusion, the OS rate of SCLC is low, and better treatment options are lacking. The exploration of SCLC subtypes and their unique therapeutic vulnerabilities may provide better insights, but further investigation is needed.

## Targeted therapy

Systematic therapy of platinum-based chemoradiotherapy has occupied the backbone role in the management of SCLC for decades. Currently, extensive research has been conducted in the field of targeted therapy for SCLC, focusing on the abnormal signaling pathways involved in cell cycle and DNA damage and repair (DDR), epigenetic regulation, cell metabolism, and tumor immunity. Despite the exploration of numerous drugs targeting various pathways for treating SCLC, only ICIs have demonstrated satisfied efficacy. Subsequently, we will present a comprehensive review for these studies. Due to the profound significance and unique characteristics of immunotherapy, it will be discussed in detail as a separate topic. Key completed and ongoing trials targeting various pathways, including the cell cycle and DDR pathway, epigenetics, metabolism, as well as NOTCH pathway and apoptosis, are shown in Tables 2 and 3, categorized by target and agent.

**Table 2:** Key completed trials for SCLC categorized by target

Study	Phase	Line of therapy	Arms	No	ORR (%)	DoR (mon)	PFS (mon)	OS (mon)
PARP								
NCT03009682 NCT03428607	2	Relapsed SCLC	Olaparib monotherapy	15	6.7	NR	1.4	8.6
			Olaparib + Ceralasertib	26	3.8	NR	2.8	7.2
STOMP	2	Chemosensitive SCLC (Maintenance after first-line therapy)	Olaparib bid	73	NR	NR	3.7	11
			Olaparib tid	73	NR	NR	3.6	9.6
			Placebo	74	NR	NR	2.5	9.7
NCT02446704	1/2	Relapsed SCLC	Olaparib + Temozolomide	48	41.7	4.3	4.2	8.5
MEDIOLA NCT02734004	1/2	Relapsed SCLC	Olaparib + Durvalumab	38	10.5	3.6	2.4	7.6
NCT02484404	2	Relapsed SCLC	Olaparib + Durvalumab	19	10.5	NR	1.8	4.1
NCT01286987	1	NR	Talazoparib monotherapy	23	8.7	3.2	2.6	NR
NCT02289690	2	Treatment-naïve ES-SCLC	Veliparib + Carboplatin + Etoposide → Veliparib	61	77	4.7	5.8	10.1
			Veliparib + Carboplatin + Etoposide → Placebo	59	59	4.3	5.7	10
			Placebo + Carboplatin + Etoposide → Placebo	61	64	5.3	5.6	12.4
ECOG-ACRIN2511 NCT01642251	2	Treatment-naïve ES-SCLC	Cisplatin + Etoposide + Veliparib	64	71.9	NR	6.1	10.3
			Cisplatin + Etoposide + Placebo	64	65.6	NR	5.5	8.9
NCT01638546	2	Relapsed SCLC	Temozolomide + Veliparib	55	39	4.6	3.8	8.2
			Temozolomide + Placebo	49	14	3.7	2	7
ZL-2306–005 NCT03516084	3	Platinum-responsive ES-SCLC	Niraparib as maintenance	125	NR	NR	1.5	9.9
			Placebo	60	NR	NR	1.4	11.4
NCT04041011	1	Relapsed SCLC	Fluzoparib + SHR-1316	16	6.3	NR	1.4	5.6
ATR								
NCT03896503	2	Relapsed SCLC	Berzosertib + Topotecan	40	26	9.3	3.9	8.9
			Topotecan	20	6	3.3	3	5.4
NCT02487095	2	Relapsed SCLC	Berzosertib + Topotecan	25	36	6.4	4.8	8.5
CHK1								
NCT02735980	2	Platinum-sensitive ES-SCLC	Prexasertib	58	5.2	5	1.4	5.4
		Platinum-resistant/Platinum-refractor ES-SCLC		60	0	0	1.4	3.2
WEE1								
NCT02937818	2	Relapsed SCLC	Adavosertib + Carboplatin	10	0	0	2.6	4.7
AURKA/B								
NCT02038647	2	Relapsed SCLC	Alisertib + Paclitaxel	89	22	3.2	3.3	6.1
			Paclitaxel	89	18	2.8	2.2	5.4
NCT01045421	2	Relapsed SCLC	Alisertib	48	21	4.1	2.1	NR
CDK4/6								
NCT04902885	3	Treatment-naïve or previously treated ES-SCLC	Trilaciclib before Etoposide/Carboplatin or Topotecan	41	44.7	4.4	4.8	12
			Placebo before Etoposide/Carboplatin or Topotecan	42	39.5	4.4	4.3	8.8
NCT03041311	2	Treatment-naïve ES-SCLC	Trilaciclib before Carboplatin/Etoposide/Atezolizumab	52	56	5.6	5.9	12
			Placebo before Carboplatin/Etoposide/Atezolizumab	53	63.5	4.3	5.4	12.8
NCT02514447	2	Relapsed SCLC	Trilaciclib before Topotecan	32	16.7	6.8	4.2	6.2
			Placebo before Topotecan	29	23.1	4.9	4.2	6.5
NCT02499770	2	Treatment-naïve ES-SCLC	Trilaciclib before Etoposide/Carboplatin	39	66.7	5.7	6.2	10.9
			Placebo before Etoposide/Carboplatin	38	56.8	5.4	5	10.6
NCT02161419	2	Treatment-naïve ES-SCLC	Roniciclib + Cisplatin/Carboplatin + Etoposide	70	60.6	4.2	4.9	9.7
			Placebo + Cisplatin/Carboplatin + Etoposide	70	74.6	4.2	5.5	10.3
HDAC								
NCT01222936	2	Relapsed SCLC	Panobinostat monotherapy	21	0	0	1.4	NR
BCL-2								
NCT00062010	2	Relapsed SCLC	Interferon alpha + 13-CRA modulation of BCL-2 + Paclitaxel	37	9	NR	2	6.2
NCT00682981	2	Chemotherapy-naïve ES-SCLC	Carboplatin + Etoposide + Obatoclax Mesylate	77	62	4.6	5.8	10.5
			Carboplatin + Etoposide	78	53	4.5	5.2	9.8
NCT00521144	2	Relapsed SCLC	Obatoclax Mesylate + Topotecan	9	0	0	2.8	NR
NCT00017251	1	Chemotherapy-naïve ES-SCLC	G3139 + Carboplatin + Etoposide	16	86	5	5.9	8.6
NCT00005032	1	Relapsed SCLC	G3139 + Paclitaxel	12	0	0	NR	NR
CALGB 30103	2	Chemotherapy-naïve ES-SCLC	Carboplatin + Etoposide + G3139	41	61	NR	6	8.6
			Carboplatin + Etoposide	15	60	NR	7.6	10.6
NCT00445198	2	Relapsed SCLC	ABT-263	39	2.6	NR	1.5	3.2
NCT00773955	2	Relapsed SCLC	AT-101	15	0	NR	1.7	8.5
NOTCH								
TAHOE NCT03061812	3	Relapsed/Refractory SCLC	Rovalpituzumab Tesirine	296	14.6	3.5	3	6.3
			Topotecan	148	20.9	4.9	4.3	8.6
NCT01901653	1	Relapsed SCLC	Rovalpituzumab Tesirine	74	18	5.6	3.1	4.6
TRINITY NCT02674568	2	Third-Line and beyond (3L +)	Rovalpituzumab Tesirine	339	12.4	4	3.5	5.6
MERU NCT03033511	3	Chemosensitive SCLC (Maintenance after first-line therapy)	Rovalpituzumab Tesirine	372	9	NR	3.7	8.8
			Placebo	376	4	NR	1.4	9.9
DeLLphi-301 NCT05060016	2	Previously treated ES-SCLC	Tarlatamab 100 mg-dose	88	32	NR	3.9	NR
			Tarlatamab 10 mg-dose	100	40	NR	4.9	NR
NCT01859741	2	Treatment-naïve ES-SCLC	Placebo + Etoposide + Cisplatin /Carboplatin	72	70.9	NR	NR	NR
			OMP-59R5 + Etoposide + Cisplatin /Carboplatin	73	68.5	NR	NR	NR

*ORR* objective response rate, *DoR* duration of response, *OS* overall survival, *PFS* progession-free survival, *NR* not reported

**Table 3:** Ongoing clinical trials for SCLC categorized by target and agent

Targets	Agents	Trial phase	ClinicalTrials.gov ID(s)
PARP	Olaparib	I, II, III	NCT02498613, NCT03532880, NCT05975944, NCT02769962, NCT04538378, NCT03923270, NCT05245994, NCT04728230, NCT05623319
	Talazoparib	I, II	NCT03672773, NCT04170946, NCT04334941
	Veliparib	I	NCT03227016
	Niraparib	I, II	NCT05718323, NCT04701307, NCT05162196, NCT03830918, NCT03221400
	Fluzoparib	I, II	NCT04933175, NCT04659785, NCT04782089, NCT04400188
	Rucaparib	II	NCT03958045
	AZD5305	I	NCT04644068
	RP12146	I	NCT05002868
ATR	Berzosertib (M6620)	I, II	NCT04768296, NCT02595931, NCT04802174, NCT04826341
	Ceralasertib (AZD6738)	II	NCT04699838
	Elimusertib (BAY 1895344)	I	NCT04514497, NCT04491942
WEE1	Debio-0123	I	NCT05815160
AURKA/B	Alisertib (MLN8237)	II	NCT06095505
	JAB-2485	I	NCT05490472
CDK4/6	Trilaciclib	II, IV	NCT05874401, NCT05578326
	Abemaciclib	II	NCT04010357
LSD1	CC-90011	I	NCT03850067
	Bomedemstat	I, II	NCT05191797
EZH2	DS-3201b	I, II	NCT03879798
	PF-06821497	I	NCT03460977
	XNW5004	I, II	NCT06022757
BCL-2	APG-1252	I	NCT04893759
Glucose metabolism	Metformin	II	NCT03994744
Arginine	ADI-PEG 20	I, II	NCT05616624, NCT03371979
NOTCH	Tarlatamab	I, III	NCT05740566, NCT06117774, NCT04885998, NCT06211036, NCT03319940, NCT05361395
	PT217	I	NCT05652686
	HPN328	I, II	NCT04471727
	BI 764532	I, II	NCT05882058, NCT04429087, NCT05879978, NCT05990738, NCT06077500
	DLL3-CAR-NK	I	NCT05507593
	DLL3-CAR-T	I	NCT05680922
	89Zr-DFO-SC16.56	I	NCT04199741

### Cell cycle and DNA damage and repair

The frequent loss of *RB1* and *TP53* in SCLC renders this tumor more vulnerable to DNA damage, thus leading to the upregulation of mediators involved in cell cycle control and the DDR pathway to maintain genomic instability and evade cell death [102]. Inhibition or loss of DDR proteins exacerbates the accumulation of DNA damage and increases the susceptibility of SCLC to various agents that cause DNA damage [103]. Therefore, DDR proteins such as PARP, ATR, CHK1 and WEE1 have been identified as potential targets for SCLC treatment.

#### Poly (ADP-ribose) polymerase, PARP

PARP is a prominent drug target among DDR proteins [104]. PARP inhibitors impede DNA repair and synergize with drugs that induce DNA damage. Additionally, PARP inhibition significantly upregulates PD-L1 expression and augments the antitumor effect of ICIs through the STING-mediated immune pathway [105, 106]. Thus, extensive investigations have been conducted to evaluate the addition of PARP inhibitors to standard chemoradiotherapy, immunotherapy, or other DNA damage agents for the treatment of SCLC [107]. Currently, PARP inhibitors that have been utilized in clinical trials for SCLC mainly include olaparib, talazoparib, veliparib, niraparib, fluzoparib, rucaparib, AZD5305 and RP12146.

PARP inhibitors are primarily used as subsequent therapeutic options after the initial treatment of extensive-stage SCLC (ES-SCLC). The efficacy of PARP inhibitor monotherapy as maintenance therapy is limited, resulting in relatively few studies [108–111]. The combination of temozolomide (TMZ) with the PARP inhibitors veliparib or olaparib was investigated in relapsed SCLC patients in two phase 2 trials. The results revealed that the addition of PARP inhibitors enhances the antitumor effect of TMZ, but no significant OS benefit has been observed (OS 8.2 months vs. 7.0 months, P = 0.50) [112, 113]. The efficacy of olaparib combined with durvalumab was tested in two single-arm trials involving relapsed ES-SCLC patients, but these trials did not meet their primary efficacy endpoints [114, 115]. PARP inhibitors have also been investigated as first-line treatment regimens for SCLC. In two phase 2 trials, the addition of veliparib to platinum-based frontline chemotherapy improved progression-free survival (PFS) in treatment-naïve patients with ES-SCLC [116, 117].

The combination of chemotherapy and immunotherapy has emerged as the recommended first-line treatment for ES-SCLC [4, 118]. In this setting, the addition of PARP inhibitors is currently being investigated in several ongoing clinical trials (NCT05245994, NCT04728230, and NCT04624204). Given the diverse responses observed in finished trials, it is important to identify patients who may benefit from PARP inhibitors. Focusing on patients who are sensitive to chemotherapy seems to be a promising approach (NCT03923270, NCT05162196, NCT03830918, NCT04782089, and NCT03958045). In addition, biomarkers play a significant role in predicting the response to PARP inhibitors. Increased sensitivity to PARP inhibitors is associated with increased SLFN11 expression [112, 119–121], decreased EMT scores and E-cadherin levels [122], decreased DNA-PKcs expression [123], increased E2F1 expression [102], decreased ATM expression [122] and the use of fluorinated [18F]-radiolabeled PARPis [124]. However, among these biomarkers, only SLFN11 has been applied for patient selection in ongoing trials (NCT05718323, NCT04334941).

In general, as valuable drugs targeting the DDR pathway, PARP inhibitors exacerbate tumor susceptibility by inhibiting DNA repair, aggravating DNA damage and enhancing tumor immunity, thereby achieving promising efficacy in first-line treatment of SCLC. In the future, guided by subtype-specific therapy, ASCL1-subtype with high levels of SLFN11 may represent a prioritized population for PARP inhibitors.

#### Ataxia telangiectasia and Rad3-related protein, ATR

As a crucial component of DDR proteins, ATR plays a pivotal role in sensing DNA damage and preserving genomic instability [125, 126]. Upon activation by DNA damage, ATR stops cell progression to the G_2_ phase through the G_2_/S checkpoint, thereby preventing cell apoptosis [127]. Three ATR inhibitors, berzosertib (M6620), ceralasertib (AZD6738) and elimusertib (BAY1895344), have been investigated in SCLC. The combination of ATR inhibitors with DNA TOP1 inhibitors was found to augment their antitumor efficacy and potentially improve the response to immunotherapy in SCLC characterized by low expression of the STING pathway [101, 128]. In single-arm trials, berzosertib combined with topotecan achieved good tolerance and critical clinical benefit in relapsed platinum-resistant SCLC patients [101, 129]. Recently, a two-arm phase 2 trial demonstrated that berzosertib plus topotecan did not improve PFS (HR = 0.80 [95% CI 0.46–1.41]; P = 0.44) in relapsed SCLC patients compared to topotecan alone, but it significantly prolonged OS (HR = 0.53 [95% CI 0.29–0.96]; P = 0.03) [130]. Two trials (NCT03428607, NCT02937818) investigated the combination of AZD6738 and olaparib in relapsed or refractory SCLC patients; however, neither achieved the predetermined therapeutic endpoint [108].

Ongoing trials are currently investigating the safety and efficacy of berzosertib combined with topotecan (NCT04768296), irinotecan (NCT02595931), or sacituzumab govitecan (NCT04826341) in relapsed SCLC patients. A previous study identified an ATR inhibitor as the most effective agent for potentiating lurbinectedin in SCLC [131]. Further clinical trials are underway to confirm its safety and efficacy in relapsed SCLC patients (NCT04802174). The addition of AZD6738 to immunotherapy as a second/third-line treatment (NCT04361825) or chemoimmunotherapy as a first-line treatment (NCT04699838) is under investigation in single-arm trials. The results of an epigenome-wide DNA methylation analysis suggested that sensitivity to ATR inhibitors may be correlated with genomic methylation levels and TREX1 expression [132]. To date, no biomarker-guided trial has been identified.

Overall, ATR inhibitors exert anti-tumor effects by promoting apoptosis induced by DNA damage and enhancing tumor immunity. The combination of ATR inhibitors with topotecan, lurbinectedin or immunotherapy has potential advantages in relapsed SCLC. However, further evidence is needed to better understand the role of ATR inhibitors in subtype-specific therapy.

#### Checkpoint kinase 1, CHK1

CHK1 is a serine/threonine protein kinase involved in DNA damage-induced cell cycle arrest and is considered as a potential therapeutic target for SCLC [133–135]. Currently, the CHK1 inhibitors utilized in the treatment of SCLC patients include prexasertib (LY2606368) and SRA-737. In both SCLC cells and mouse models, promising antitumor efficacy has been achieved by CHK1 inhibitor monotherapy or in combination with chemotherapy or PARP inhibition. These findings highlight the potential of CHK1 inhibitors to overcome resistance to chemotherapy or PARP inhibitors [99, 136–138]. In addition, CHK1 inhibition activates the function of cytotoxic T lymphocytes via the innate immune STING pathway and enhances the antitumor effect of ICIs [105].

The recommended dose of LY2606368 monotherapy was established in a phase 1 trial, but a subsequent phase 2 trial failed to achieve the anticipated efficacy in platinum-resistant ES-SCLC patients [139, 140]. Another oral CHK1 inhibitor, i.e., SRA-737, was tested in a phase 1/2 trial. The combination of SRA-737 and low-dose gemcitabine resulted in a partial response rate of 11.1% (1/9) in SCLC patients [141]. Furthermore, the addition of SRA737 and low-dose gemcitabine enhances the antitumor efficacy of PD-L1 blockade, highlighting a potential triple combination therapy [142]. The inhibition of WEE1 reversed LY2606368 resistance in SCLC cell lines, thereby providing evidence for the synergistic potential of CHK1 and WEE1 inhibitors [143].

To sum up, CHK1 inhibitors impede DNA damage repair, resulting in the formation of replication barriers and induction of apoptosis in cancer cells. However, the efficacy of CHK1 inhibitors for SCLC remains unsatisfactory. The overexpression of MYC has been identified as a candidate biomarker for CHK1 inhibitors [99, 144]. Thus, despite suboptimal performance, CHK1 inhibitors may be applicable to NEUROD1 and other ASCL1-low subtypes characterized by MYC overexpression.

#### WEE1

WEE1 is a protein tyrosine kinase that inactivates cyclin-dependent kinase (CDK) 1/2 in the cell cycle and protects against DNA replication through the regulation of histone synthesis and epigenetic modification [145–147]. The combination of a WEE1 inhibitor and a PARP inhibitor exhibits promising antitumor efficacy within circulating tumor cell (CTC)-derived explant SCLC models [148, 149]. Inhibition of WEE1 promotes the immune response via the STING-TBK1-IRF3 pathway, enhances the antitumor effect of PD-L1 antibodies through the STAT1 pathway, and significantly suppresses tumor progression in SCLC models (including MYC-stabilized SCLC) [150].

Adavosertib (AZD1775) is an oral WEE1 inhibitor that has been tested in several advanced solid tumors. In a three-arm trial (NCT02937818), the efficacy of AZD1775 in combination with carboplatin was evaluated in platinum refractory ES-SCLC patients, with a median OS of 4.67 months, indicating the potential efficacy in SCLC. An ongoing trial is investigating the safety and efficacy of the novel WEE1 inhibitor Debio-0123 in combination with etoposide and carboplatin in patients with relapsed SCLC (NCT05815160).

In summary, WEE1 does not appear to be a worthy target in the treatment of SCLC, as the efficacy of WEE1 inhibitors is quite limited and its potential has yet to be fully explored in subtype-specific therapy.

#### Aurora kinase A/B, AURKA/B

The aurora kinase family is classified as serine/threonine kinases that play crucial roles in regulating the G_2_/M transition and spindle assembly checkpoint during the cell cycle [151]. *RB1* gene mutations and *MYC* overexpression or amplification frequently occur in SCLC, leading to high sensitivity to AURKA/B inhibitors [39, 91, 144, 152–154]. A recent study demonstrated the potential of AURKA/B inhibitors to augment the antitumor efficacy of PD-L1 blockade via the restoration of inflammatory gene expression [155]. Currently, three AURKA inhibitors, namely, alisertib (MLN8237), JAB-2485, and erbumine (LY3295668), are used to treat SCLC.

A multicenter phase 1/2 trial investigated the efficacy of the oral AURKA inhibitor alisertib as monotherapy in advanced tumors. Among 48 relapsed SCLC patients, 10 (20.8%) achieved an objective response, and a median FPS of 2.1 months was observed [156]. In a separate phase 1 trial, alisertib was combined with nab-paclitaxel, resulting in a partial response in 1 out of 5 refractory SCLC patients [157]. In a phase 2 trial, the combination of alisertib and paclitaxel as a second-line treatment demonstrated superior PFS benefits for patients with relapsed SCLC expressing MYC compared to monotherapy with paclitaxel (PFS 4.6 months vs. 2.3 months; HR = 0.29, 95% CI 0.12–0.72) [158]. An ongoing phase 2 trial (NCT06095505) is evaluating the safety and efficacy of alisertib in progressed ES-SCLC patients who are receiving or have completed first-line treatment with chemotherapy combined with anti-PD-L1 immunotherapy. Further ongoing trial will determine the role of AURKA inhibitor.

In conclusion, AURKA inhibitors present promising antitumor efficacy through the inhibition of MYC. AURKA may be considered as a potential therapeutic vulnerability for NEUROD1, POU2F3 and Inflamed subtypes based on the transcriptional classification, and nmf4 subtype based on the multi-omics classification that exhibit overexpression of MYC in subtype-specific therapy.

#### Cyclin-dependent kinase 4/6, CDK4/6

The aberrant activation of CDK4/6 results in excessive phosphorylation of the Rb protein, leading to dysregulation of the G_1_/S transition and promoting tumorigenesis [159–161]. The antitumor effect of CDK4/6 inhibitors has not been confirmed in SCLC, but they have shown strong protective effects against chemotherapy-induced myelosuppression (CIM) [162]. The transient and reversible arrest of hematopoietic stem and progenitor cells (HSPCs) in the G_1_ phase is achieved through the inhibition of CDK4/6, thereby providing protection against cytotoxic injury induced by chemotherapy [163]. Several clinical trials have demonstrated the protective effects of the CDK4/6 inhibitor trilaciclib in CIM [164–166]. Thus, the Food and Drug Administration (FDA) has granted approval to trilaciclib for alleviating CIM in ES-SCLC patients [167]. Additionally, the incorporation of trilaciclib into chemotherapy plus ICIs effectively preserved immune system function and augmented the antitumor response in preclinical models [168].

An ongoing phase 4 trial is evaluating progression and survival in ES-SCLC patients when trilaciclib is added to topotecan-containing chemotherapy (NCT05874401). The effect of trilaciclib in combination with lurbinectedin is currently being investigated in a phase 2 trial (NCT05578326). The efficacy and safety of abemaciclib, a novel CDK4/6 inhibitor, are under evaluation in Rb wild-type refractory ES-SCLC (NCT04010357). All in all, although CDK4/6 inhibitors do not specifically target any subtypes, they hold significant and extensive implications as protective agents to alleviate the adverse effects of chemotherapy.

### Epigenetics

The accumulation of genomic structural and functional changes is widely recognized as a primary force driving cancer development. Epigenetic modification represents one such mechanism. Epigenetic modifications are characterized by heritable changes in gene activity that occur without altering the DNA sequence. Abnormal modifications regulate gene expression patterns that promote tumorigenesis and facilitate the acquisition of hallmark tumor capabilities [169]. The two primary types of epigenetic modifications are DNA methylation and histone modifications. Clinicians are leveraging these events as adjunctive tools in clinical decision-making. Furthermore, the reversibility of epigenetic modifications has led to the emergence of epigenetic therapy as a promising strategy for treating SCLC [170, 171].

#### Lysine-specific demethylase 1, LSD1

As a form of histone modification, histone methylation represents one of the critical epigenetic hallmarks of SCLC. Encoded by *KDM1A*, LSD1 functions as a histone demethylase that selectively removes monomethylated and dimethylated groups from histone H3K4 and H3K9 sites, thereby influencing gene transcription [172]. Through interaction with SNAG domain-containing proteins, namely, INSM1 and GFI1B, LSD1 facilitates the transcriptional activation of genes associated with NE phenotypes and augments the proliferation of SCLC cells. Perturbation of this interaction attenuates the expression of pivotal genes, such as ASCL1, and impedes tumor proliferation [173]. Similarly, ZFP36L1 has been identified as a target gene of LSD1, which binds to and destabilizes SOX2 and INSM1, thus regulating the NE differentiation of SCLC cells [174]. A recent study demonstrated that LSD1 inhibition activates NOTCH signaling, leading to a subsequent reduction in ASCL1 expression in SCLC [95]. Additionally, the selective LSD1 inhibitor GSK2879552 was found to induce growth inhibition in SCLC cell lines [175]. These findings underscore the potential of LSD1 as a therapeutic target for SCLC.

At present, a range of drugs targeting LSD1 have been identified, including GSK2879552, CC-90011 and bomedemstat (IMG-7289). Currently, another reversible LSD1 inhibitor, CC-90011, is being evaluated in combination with cisplatin/etoposide with or without nivolumab for untreated ES-SCLC patients (NCT03850067). The combination of LSD1 inhibitors with immunotherapy represents an innovative therapeutic strategy, as several studies have suggested that the inhibition of LSD1 enhances the antitumor efficacy of immune checkpoint blockade [176–179]. A concomitant trial is being conducted to assess the effect of bomedemstat and maintenance immunotherapy with atezolizumab in newly diagnosed ES-SCLC patients (NCT05191797). Collectively, the clinical efficacy of LSD1 remains unproven with no successful clinical trials. Two early-stage clinical trials are ongoing to verify its effectiveness. If successful, these combinations could offer new hope for improving outcomes in SCLC patients.

#### Histone deacetylases, HDACs

Apart from histone methylation, histone acetylation is another crucial epigenetic hallmark in SCLC. Histone acetylation/deacetylation modulates the transcriptional regulation of genes implicated in the initiation, progression, and metastasis of SCLC by modifying chromatin accessibility [170]. Overexpression of HDACs in cancer cells results in increased deacetylation, which adversely affects the expression of tumor suppressor genes [180]. Administration of HDACs inhibitors in murine models was observed to upregulate YAP through attenuation of the activity of the RE1-silencing transcription factor-corepressor-HDAC complex, hence suppressing metastasis and improving survival in SCLC [181]. Furthermore, a series of studies revealed that HDAC inhibitors potentiate the efficacy of conventional chemotherapeutic regimens [182, 183]. This synergistic interaction might be mediated via the induction of S-phase arrest and decreased base excision repair induced by HDAC inhibition [184].

Currently, four HDAC inhibitors (vorinostat, belinostat, panobinostat, and romidepsin) have received FDA approval for use in certain cancers. Related research is undertaken in SCLC as well. For instance, a multicenter, nonrandomized phase 2 trial evaluated the antitumor activity of panobinostat in patients with previously treated SCLC [185]. At the first assessment, more than 30% tumor shrinkage was observed in 2 of the 19 patients [185]. Another phase 1 trial assessing the combination of belinostat with cisplatin and etoposide demonstrated promising results in SCLC patients [186]. An objective response was observed in 7 (47%) of 15 patients with NE tumors (including SCLC) [186].

Overall, the existing FDA approvals for HDAC inhibitors in other cancers and the positive outcomes in early trials highlight their potential as a viable therapeutic option for SCLC. Further clinical trials are essential to establish the efficacy and safety of HDAC inhibitors in SCLC.

#### Enhancer of Zeste homolog 2, EZH2

EZH2 is a pivotal oncogene linked to methylation processes in SCLC. Specifically, EZH2 is the core component of polycomb repressive complex 2 (PRC2), a histone methyltransferase responsible for methylating lysine at position 27 on histone H3 (H3K27me3), which is crucial for maintaining epigenetic gene silencing [187]. EZH2 regulation is mediated by the pRB-E2F axis, and its expression is frequently augmented in SCLC as a consequence of *RB1* alterations [86, 188]. Through modulation of apoptosis and cell cycle regulation, EZH2 promotes E2F-driven SCLC tumorigenesis [189]. EZH2-mediated epigenetic modifications also lead to the upregulation of TWIST1 and the suppression of SLFN11 in SCLC, contributing to resistance to chemotherapy [190]. Moreover, the growth and chemoresistance of SCLC cells have been proven to be mediated by TUG1, which regulates LIMK2b via EZH2 [191].

Hence, therapeutic interventions targeting EZH2 might be beneficial for SCLC patients. Currently, the EZH2 inhibitor PF-06821497 is under clinical investigation as a monotherapy in patients with relapsed or refractory SCLC (NCT03460977). Based on the correlations between EZH2 and chemoresistance, a phase 1/2 trial (NCT03879798) is assessing the safety and efficacy of the EZH1/2 inhibitor DS-3201b in combination with irinotecan for patients with recurrent SCLC. Moreover, the overexpression of EZH2 may contribute to radioresistance [192], suggesting that patients with radioresistant SCLC could also benefit from EZH2 inhibition. Furthermore, a correlation between EZH2 expression and the response to immunotherapy has been identified in various types of cancer [193], indicating that combining EZH2 inhibitors with immunotherapy may enhance treatment efficacy. The phase 1b/2 KEYNOTE F19 trial (NCT06022757) is investigating the role of XNW5004 in combination with pembrolizumab for relapsed SCLC patients.

Totally, albeit the clinical application of EZH2 inhibitors in SCLC is still being explored. The diverse mechanisms by which EZH2 inhibition may enhance treatment efficacy through overcoming chemoresistance, radioresistance, and improving immunotherapy response, suggest that EZH2 is a promising target for SCLC therapy.

### Other pathways

#### Metabolism

The relentless pursuit of effective treatments for SCLC has led to a focus on the metabolic underpinnings of this disease. The rapid growth and progression of cancer are sustained by altered metabolic pathways, including the way cells process glucose and amino acids such as arginine. Such metabolic alterations not only fuel cancer growth but also offer potential targets for therapy. These vulnerabilities are being exploited through various pathways, aiming to cut off the cancer's energy supply and building blocks necessary for its growth.

##### Glucose metabolism pathway

Glycolysis, oxidative phosphorylation, and the pentose phosphate pathway are three primary branches of glucose metabolism [194]. Glycolysis, which is prominent in the Warburg effect, is the major source of energy for cancer cells. This metabolic reprogramming allows cancer cells to produce high energy levels even under anaerobic or hypoxic conditions at the expense of high glucose intake. Hence, elevated glucose uptake is often observed in most SCLC patients and is associated with a poor prognosis [195, 196]. Elevated glycolysis, aligning with the Warburg effect, has been detected in cell lines overexpressing MYC [197]. Administration of the glycolysis inhibitor PFK158 in xenograft models led to delayed tumor progression and a reduction in the expression of genes associated with glycolysis [197]. Currently, both preclinical and clinical trials are being performed to test drugs that interfere with glucose metabolic pathways or downstream molecules. For instance, the antidiabetic drug metformin was found to improve both the OS and PFS of diabetic CSCLC patients (OS 19.0 vs 11.5 months, p < 0.001; DFS 10.5 vs 7.0 months, p < 0.001) [198]. Additionally, metformin may reverse acquired resistance to PD-1 inhibitors in SCLC [199]. A phase 2 trial (NCT03994744) is evaluating the safety and efficacy of combining metformin with sindilizumab, a PD-1 inhibitor, in pretreated ES-SCLC patients. Despite their promising therapeutic effects, metabolism-based therapies may encounter challenges such as nonspecific toxicity [200, 201]. Many obstacles still need to be overcome in this field.

##### Arginine

As the precursor for polyamine biosynthesis, NO generation and mTOR pathway activation, arginine plays a vital role in multiple cellular physiological processes. A key enzyme in the synthesis of arginine is ASS1, the expression of which is often reduced in SCLC. Loss of ASS1 causes notable bioenergetic alterations in SCLC, resulting in arginine dependence, which is correlated with chemoresistance and poor clinical outcomes [98, 202–204]. Treatment with pegylated arginine deiminase (ADI-PEG 20) to induce arginine depletion markedly impeded tumor growth and enhanced the survival of mice bearing MYC-driven tumors [98]. Hence, arginine deprivation may serve as a subtype-specific therapeutic vulnerability for patients with SCLC.

Currently, a therapeutic regimen comprising ADI-PEG 20 in combination with gemcitabine and docetaxel is under active clinical investigation for SCLC patients who progressed after frontline therapy (NCT05616624). Concurrently, the potential synergistic effect of ADI-PEG 20 administered in combination with pembrolizumab (NCT03371979) has also been explored in a phase 1 trial. However, the outcomes of these studies have not yet been reported. These efforts suggest a promising direction, with two ongoing clinical trials exploring ADI-PEG20 combinations, but it is clear that arginine-targeted therapies still have a considerable journey ahead in proving their efficacy.

#### NOTCH pathway

Comprising NOTCH receptors, DSL family ligands, and numerous signal transduction molecules, the NOTCH signaling pathway orchestrates several cellular functions: cell proliferation, stem cell maintenance, differentiation, and apoptosis [205]. Alterations that result in the loss of NOTCH signaling function have been frequently observed among SCLC patients [16, 206]. Previous studies have also revealed that NOTCH signaling is implicated in chemoresistance and modulation of the immune microenvironment, thus underscoring its potential as an antitumor target in SCLC [16, 206].

DLL3 is a single-pass type I transmembrane protein and a member of the inhibitory ligands of the NOTCH pathway. DLL3 interacts with NOTCH receptors, exerting inhibitory effects on the NOTCH pathway in SCLC. DLL3 is overexpressed in SCLC and certain neuroendocrine tumors, whereas its expression is minimal in healthy individuals [207]. This expression pattern has sparked significant interest in developing DLL3 as a novel therapeutic target for SCLC and other malignancies [207].

Rova-T is an antibody‒drug conjugate that targets DLL3 with a specialized humanized monoclonal antibody. The initial human study by Rova-T reported an objective response rate (ORR) of 18% in previously treated SCLC patients [93]. However, the phase 2 TRINITY study reported that grade 3 to 5 adverse events (AEs) were seen in 213 (63%) patients in the third-line and beyond settings [94]. The phase 3 TAHOE and MERU trials, which evaluated Rova-T with topotecan as second-line therapy and Rova-T as maintenance therapy after first-line treatment, were both halted early due to failure of predetermined PFS and OS [208, 209].

Tarlatamab (AMG 757) is a bispecific T-cell engager that targets dual DLL3 and CD3 [210]. A promising response durability was observed in SCLC patients treated with tarlatamab monotherapy, with a reported ORR of 23.4% (95% CI 15.7–32.5) [211]. Recently, the phase 2 DeLLphi-301 trial (NCT05060016) demonstrated persistent antitumor activity of tarlatamab in patients with relapsed/refractory SCLC. Compared to patients in the 100 mg dose group, patients in the 10 mg dose group had superior outcomes, with an objective remission rate of up to 40% (97.5% CI 29–52), as opposed to 32% (97.5% CI 21–44) [212]. To compensate for the absence of a standard treatment control group, the phase 3 DeLLphi-304 study (NCT05740566) will compare the efficacy of tarlatamab with that of the standard care in relapsed SCLC patients. Additionally, the efficacy of tarlatamab after chemoradiotherapy in patients with limited-stage SCLC (LS-SCLC) is being evaluated in the phase III DeLLphi-306 study (NCT06117774). Additionally, the combined effects of tarlatamab and chemoimmunotherapy are being explored in multiple clinical trials. An ongoing phase 1b study (NCT04885998) is evaluating the safety and efficacy of tarlatamab in combination with AMG 404 in SCLC patients. In the phase 3 DeLLphi-305 trial (NCT06211036), tarlatamab and durvalumab versus durvalumab alone is being compared in first-line ES-SCLC following platinum, etoposide and durvalumab treatment. Its combined effect with carboplatin, etoposide, and PD-L1 inhibitors in ES-SCLC is also being investigated in a phase 1b trial (NCT05361395). Similar to tarlatamab, BI 764532 redirects T cells to eradicate tumor cells and serves as a DLL3-targeted treatment for SCLC. Two trials are testing the effects of different doses of BI 764532 monotherapy in SCLC (NCT04429087 and NCT05882058). The combination of BI 764532 with the PD-1 inhibitor ezabenlimab is being explored in SCLC patients positive for DLL3 (NCT05879978). The efficacy of different doses of BI 764532 in addition to standard treatment or topotecan are also being tested in SCLC (NCT05990738, NCT06077500). Additionally, trials are recruiting volunteers for other drugs, such as HPN328, PT217 and 89Zr-DFO-SC16.56 (NCT04199741, NCT04471727, and NCT05652686).

The efficacy of CAR-T-cell therapy in blood cancers has prompted investigations into its potential application for solid tumors. In a phase 1 trial of AMG 119 involving five relapsed/refractory SCLC patients, one patient achieved a partial response (PR) with 43% shrinkage in lesion size, and the other patients exhibited a 16% reduction in lesion size along with the resolution of several liver metastases (NCT03392064). These initial data support the continued development of DLL3 CAR-T-cell therapy for SCLC. An ongoing phase 1 trial is recruiting volunteers for further validation in patients with ES-SCLC (NCT05680922). Similarly, CAR-NK-cell therapy has also demonstrated short-term effects, with noticeable shrinkage of the remaining metastatic lesions in SCLC patients [213]. A related trial using DLL3-CAR-NK cell therapy for treating ES-SCLC is underway (NCT05507593).

Other molecules, such as NOTCH2 and NOTCH3, also represent alternative targets for modulating NOTCH signaling. Tarextumab (anti-NOTCH2/3, OMP-59R5) is a human monoclonal antibody that targets NOTCH2 and NOTCH3 receptors. A study utilizing PDX tumors preliminarily demonstrated the antitumor effect of tarextumab [214]. Subsequently, a phase 1 dose-escalation study indicated good tolerability of tarextumab in patients with advanced solid tumors [215]. However, a combined phase 1b/2 PINNACLE trial investigating tarextumab and chemotherapy in SCLC patients was terminated due to a lack of improvement in PFS (NCT01859741).

In addition to serving as a therapeutic target, NOTCH signaling mutations are associated with improved clinical benefits in SCLC patients undergoing immunotherapy. In a cohort of 662 patients receiving ICIs, the NOTCH4 mutation group had better objective remission rates, clinical benefit rates, and longer PFS and OS, indicating that NOTCH signaling is a determinant of the response to ICIs in SCLC patients [68].

Beyond doubt, the inhibitor of NOTCH signaling pathway, particularly DLL3, has shown more promising results for SCLC. As a potential marker in the multi-omics model of the nmf2 subtype, DLL3 emerges as an attractive therapeutic target. Despite challenges with Rova-T, promising results from drugs like tarlatamab and BI 764532 highlight the therapeutic promise of targeting NOTCH signaling. Further study and combination therapies may better improve prognosis of SCLC patients.

#### Apoptosis

##### BCL-2

Through the modulation of the mitochondrial outer membrane, BCL2 family proteins control the cellular decision between survival and apoptosis [216]. The overexpression of BCL2, an important member of the BCL2 family, is frequently observed in SCLC and is linked to the development of drug resistance and poor prognosis [217]. An FDA approved BCL2 inhibitor, venetoclax, was proven to block tumor growth and induce tumor regression in mice bearing high BCL2 expressing SCLC [217]. Moreover, high BCL2 levels have been demonstrated to suppress DNA damage and apoptosis induced by the AURKB inhibitor AZD2811. Resistant models could be significantly sensitized by the combination of AZD2811 with the BCL2 inhibitor venetoclax [218]. Additionally, the synergistic effect of receptor tyrosine kinase-like orphan receptor 1 inhibition with BCL2 inhibition was observed in SCLC models [219].

The combination of oblimersen (G3139), an antisense BCL2 oligonucleotide, with carboplatin and etoposide or paclitaxel was shown to be well tolerated in phase 1 trials [220, 221]. However, drugs such as obatoclax (a first-generation BCL2 inhibitor), AT-101 (a small-molecule BCL2 inhibitor), ABT-263, and isotretinoin failed to obtain clinical benefits in phase 2 trials [222–225]. The addition of oblimersen to a standard regimen also failed to improve any clinical outcomes in the phase 2 CALGB 30103 trial [226].

Of note, BCL2 may be a potential molecular target for SCLC. Despite promising preclinical results, BCL-2 inhibitors have failed in phase 2 trials. Additional clinical trials are needed to fully elucidate its therapeutic potential.

### Immunotherapy

#### Extensive-stage SCLC, ES-SCLC

SCLC is considered a good candidate for ICIs owing to its increased TMB and the presence of autoimmune paraneoplastic phenomena. Currently, several ICIs, such as atezolizumab, durvalumab, serplulimab and adebrelimab, which target the PD-1/PD-L1 pathway, have been approved for the treatment of ES-SCLC by the National Comprehensive Cancer Network (NCCN) or Chinese Society of Clinical Oncology (CSCO) guidelines. However, the process is filled with thistles and thorns.

Ipilimumab, a monoclonal antibody against CTLA4, was first used following an early paclitaxel/carboplatin-induced regimen in patients with ES-SCLC, and the immune-related PFS improved significantly compared with that in the control group [227]. CheckMate 032, a multicenter, phase 1/2 trial executed later, explored the role of nivolumab, an anti-PD-1 antibody, as well as the role of nivolumab combined with ipilimumab in previously treated SCLC patients. The final results confirmed the durable antitumor activity and manageable safety of ICIs [228]. Similar promising antitumor activity and safety of pembrolizumab, another anti-PD-1 antibody, was achieved in previously treated SCLC patients with recurrence or metastasis, as shown by KEYNOTE-028 and KEYNOTE-158 [229, 230]. However, in CheckMate 331, a randomized phase 3 trial, the advantage of nivolumab as a second-line treatment in patients with relapsed SCLC could not be verified [231]. Taken together, these clinical trials indicate that the role of ICIs in SCLC as a posterior treatment is contradictory, and the consolidated role of ICIs in different phases needs further investigation.

To explore the role of ICIs in maintenance therapy for ES-SCLC, several studies have been performed. A phase II study revealed that maintenance of pembrolizumab, a PD-1 inhibitor, in ES-SCLC patients receiving standard chemotherapy failed to improve patient prognosis compared with historical data [232]. In the CheckMate 451 study, compared with placebo, nivolumab plus ipilimumab or nivolumab monotherapy maintenance did not achieve desirable outcomes in patients with ES-SCLC following first-line chemotherapy [233]. Thus, further regimens should be administered.

Owing to the potential promising efficacy and contradictory role of ICIs, different immune-monoclonal antibodies combined with first-line chemotherapy followed by ICIs as maintenance therapy were tested in patients with ES-SCLC. The regimen of ipilimumab plus etoposide and platinum was first applied in ES-SCLC via a phase 3 randomized trial, and the outcomes showed that immunotherapy did not lead to a better OS [234]. A similar failure to improve OS was observed in the KEYNOTE-604 study, which used pembrolizumab as a first-line therapy in patients with ES-SCLC [235]. Undoubtedly, the above disappointing conclusions cast a shadow on the exploration of immunotherapy. With persistent effort, the IMpower133 randomized double-blind phase III trial revealed that the addition of atezolizumab, an anti-PD-L1 antibody, to standard chemotherapy could significantly prolong the median OS in patients with ES-SCLC for 2 months [4]. Thereafter, CASPIAN randomized phase 3 trials also confirmed that the anti-PD-L1 antibody durvalumab in combination with first-line treatment could yield positive results, with an extended median OS of 3 months in patients with ES-SCLC [236]. Accordingly, atezolizumab and durvalumab are approved as first-line immunotherapies combined with standard chemotherapy for treating ES-SCLC by the US FDA. The CAPSTONE-1 and ASTRUM-005 phase 3 trials also yielded positive results using different PD‐L1 (adebrelimab) and PD-1 (serplulimab) inhibitors, respectively, and these two drugs were approved by the National Medical Products Administration (NMPA) for first-line treatment of ES-SCLC [237, 238]. In a recent RATIONALE-312 study, another PD-1 inhibitor, tislelizumab, demonstrated superior OS and PFS when combined with chemotherapy as a first-line treatment for ES-SCLC [239].

Unsurprisingly, ICIs, as a first-line treatment, have yielded promising benefits and represent a novel treatment approach for ES-SCLC. Nonetheless, the role of ICIs combined with different regimens remains elusive. Radiotherapy combined with ICIs can exhibit synergistic effects by remodeling the tumor microenvironment (TME) [240]. However, this regimen needs further investigation via evidence-based data, such as the RAPTOR/NRG LU007 trial (NCT04402788) and LEAD trial (NCT05092412), although current guidelines suggest that thoracic radiotherapy could be used with immunotherapy [241]. The combination of ICIs with targeted therapies may have promising applications. The phase II PASSION study evaluated the PD-1 inhibitor camrelizumab plus the antiangiogenic drug apatinib in patients with ES-SCLC. The results showed that the above treatment significantly enhanced PFS in ES-SCLC patients who did not respond to first-line platinum-based chemotherapy, with a favorable safety profile [242]. Moreover, the phase 3 ETER701 trial demonstrated that the addition of the PD-L1 inhibitor TQB2450 and anlotinib to standard chemotherapy significantly improved the OS and PFS of patients with ES-SCLC [243]. For the role of two ICIs in ES-SCLC, different regimens should be used. However, the SKYSCRAPER-02 trial revealed that atezolizumab combined with extra tiragolumab (an anti-TIGIT monoclonal antibody) did not significantly improve PFS or OS compared to that of the control group [244].

#### Limited-stage SCLC, LS-SCLC

Although immunotherapy has achieved remarkable success in patients with ES-SCLC, its efficacy in patients with LS-SCLC remains unconfirmed. Two single-arm phase 1 trials exploring the efficacy of chemoradiotherapy (CRT) combined with duvarizumab [245] or pembrolizumab [246] in treating LS-SCLC achieved 2-year survival rates of 67.8 and 65.8%, respectively. Despite the promising clinical efficacy and tolerable toxicity observed in single-arm trials, consolidation therapy with ICIs following concurrent CRT (CCRT) failed to improve the survival of patients with LS-SCLC in the STIMULI trial [247]. The phase 2 STIMULI trial was designed to explore the efficacy and safety of nivolumab in combination with ipilimumab as maintenance therapy for patients with LS-SCLC who have not progressed after receiving CRT and prophylactic cranial irradiation (PCI) [247]. No significant improvement in PFS was observed, potentially due to drug toxicity reactions that limit the number of patients able to receive maintenance therapy [247].

Further investigations are currently underway to investigate the efficacy of combining ICIs with CRT. The phase 3 ADRIATIC trial (NCT03703297) demonstrated that durvalumab with or without tremelimumab after concurrent CRT significantly improved the PFS and OS of patients with LS-SCLC [248, 249]. The safety run-in results of a phase 3 study (NCT04691063) revealed that SHR-1316 combined with concurrent chemoradiotherapy achieved promising clinical efficacy and tolerable safety [250]. A subsequent randomized, double-blind and placebo-controlled study is currently ongoing. Similarly, two phase 2 trials (NCT03540420 and NCT03811002) of atezolizumab have also completed recruitment, potentially confirming the role of immunotherapy in LS-SCLC.

#### Emerging targets for immunotherapy

CD47 is highly expressed on the surface of SCLC cells and interacts with signal-regulatory protein alpha (SIRPα) receptors on macrophages, thereby inhibiting phagocytic activity and facilitating immune evasion [251, 252]. Thus, antibodies targeting the CD47/SIRPα axis can activate macrophages and enhance antitumor immunity [252–254]. Recent studies have demonstrated that CD47 inhibitors augment the therapeutic efficacy of local radiotherapy and exert distant effects by suppressing the growth of nonirradiated tumors [255]. PT217, a bispecific antibody targeting DLL3 and CD47, is under investigation among patients with SCLC and other NE cancers in the phase 1 SKYBRIDGE study (NCT05652686).

The immune checkpoint B- and T-lymphocyte attenuator (BTLA), which is detected at high levels on T and B lymphocytes, dendritic cells and macrophages, can interact with herpesvirus entry mediator (HVEM) expressed on tumor cells and T and B lymphocytes, NK cells and myeloid cells [256]. The BTLA/HVEM signaling pathway is negatively associated with the immune response via the recruitment of phosphatases 1 and 2 [257]. An emerging study demonstrated that treatment with an anti-BTLA antibody (tifcemalimab) combined with toripalimab and chemotherapy has tolerable side effects in patients with ES-SCLC, with an 86.5% ORR and a 100% disease control rate (NCT05000684) [258]. The role of tifcemalimab in LS-SCLC is also being investigated (NCT06095583).

As an immune checkpoint regulator, B7 homolog 3 protein (B7-H3) modulates T-cell activation through its costimulatory and coinhibitory roles, making it a promising target for SCLC treatment [259, 260]. B7-H3 is overexpressed in SCLC and has been linked to unfavorable outcomes [261, 262]. Ifinatamab deruxtecan (I-DXd) is an antibody‒drug conjugate that targets B7-H3 and delivers the topoisomerase I inhibitor deruxtecan. This drug is being evaluated in several ongoing trials. DS7300-A-J101 (NCT04145622) is a phase I/II clinical trial that enrolled patients with advanced, unresectable or metastatic solid tumors. In a subgroup of 21 SCLC patients, the study reported an ORR of 52.4%, with a complete response (CR) rate of 4.8% and a median OS of 12.2 months. In the phase 2 IDeate-Lung01 trial (NCT05280470), the efficacy, safety and pharmacokinetics of I-DXd were investigated in pretreated ES-SCLC patients. The recently initiated phase 1b/2 IDeate-Lung03 trial (NCT06362252) aimed to assess the efficacy of I-DXd plus atezolizumab, with or without chemotherapy, as first-line induction or maintenance therapy in patients with ES-SCLC.

Natural Killer Group 2A (NKG2A) is an inhibitory receptor found on the surface of both T and NK cells [263]. The inhibition of NKG2A unleashes the function of T and NK cells and promotes antitumor immunity [264]. The efficacy of the NKG2A inhibitor monalizumab combined with durvalumab plus platinum-based chemotherapy has been evaluated in a single-arm phase II MOZART trial (NCT05903092).

In addition to ICIs, the initiation of innovative immunotherapy based on the infusion of immune cells, such as dendritic cells (DCs) and cytokine-induced killer (CIK) cells, has gained considerable attention. In a phase 2 trial, a vaccine (Ad.p53-DC) containing dendritic cells transfected with wild-type TP53 failed to improve the response to chemotherapy in recurrent ES-SCLC patients, but its safety and therapeutic immune potential remain encouraging [265]. However, a subsequent trial (NCT03406715) of the Ad.p53-DC vaccine combined with ipilimumab and nivolumab was terminated, and the results were limited. Additionally, the combination of CIK cell transfusion and chemotherapy has shown promise with a 4-month PFS [266]. Maintenance therapy with the PD-1 inhibitor sintilimab after first-line CIK cell therapy plus chemotherapy also presented satisfactory safety and antitumor efficacy [267]. Thus, standard immunochemotherapy combined with immune cell therapy seems to be a promising strategy.

Virotherapy is an emerging field in the treatment of lung cancer. Oncolytic viruses are a class of viral agents capable that selectively target neoplastic cells and augment the antitumor immune response [268]. Two types of viruses, namely, Seneca Valley virus (NTX-010) [269] and a modified oncolytic myxoma virus (MYXV) [270], have been used in SCLC trials. Although a phase 2 trial revealed that ES-SCLC patients did not benefit from NTX-010 treatment following platinum-based chemotherapy, the potential of viroimmunotherapy remains promising [269]. Another oncolytic virus, RT-01, is currently being evaluated in a single-arm phase 1 trial (NCT05205421) among ES-SCLC patients.

## Conclusion and future directions

SCLC, closely correlated with heavy smoking, is considered as a recalcitrant cancer. Despite the unambiguous analysis of genomic alterations, no valuable targeted therapy analogous to adenocarcinoma with genomic alterations has been confirmed for SCLC. Due to treatment limitations and the intrinsic aggressive features of SCLC, patients still have poor outcomes. Systematic exploration of SCLC subtypes and signaling pathways may provide novel insight for SCLC treatment. Based on diverse transcriptional data, different SCLC subtypes and specific treatments for these subtypes were identified. Integrated multi-omics analysis revealed four novel subtypes of SCLC, which may provide insight into therapeutic regimens. In addition to the classification of SCLC, investigations of abnormal signaling pathways (mainly those involved in the cell cycle and DNA damage and repair, epigenetics, metabolism and others) can also lead to progress in treatment. However, many problems still exist, and further attention should be devoted to these issues.

Based on different data and method used for classification, diverse subtypes were shown. Yet, uniform and rigorous consensus clustering of SCLC patients is still under debate, not to mention the biomarker for each subtype. For classic ASCL1, NEUROD1, YAP1 and POU2F3 subtypes, immunohistochemistry can be used. However, low levels of YAP1 coexisted with other subtypes, and high rate of coexpression of ASCL1 and NEUROD1 can be detected. In terms of ASCL1, NEUROD1, POU2F3 and Inflamed subtypes, they can largely be defined by differential level of ASCL1, NEUROD1, POU2F3, and low expression of these three factors. With regard to multi-omics subtypes, nmf4 and nmf2 can be mostly defined by POU2F3 and DLL3 expression, the rest of them are still lack of specific biomarker. And further efforts are still needed to solve the clustering and biomarkers.

The low number of tumor samples has blocked the exploration of SCLC. The high quality of samples from clinical trials can better illustrate the treatment response at the molecular level. For instance, transcriptional data from IMpower133 revealed that patients with the Inflamed subtypes can benefit greatly from immunotherapy. Thus, clinical trials with the support of samples could promote the progression of SCLC.

The bank of the PDX/CDX model can not only serve as a tool for basic study and treatment, but also provide adequate tissues for multi-omics, which can offer additional information for SCLC. However, the complicated process, high prices and low rate of model development limit progress, and further efforts should be made.

Fewer drugs have been approved for SCLC in recent decades. With the development of pharmaceutical technology, including the use of ADCs and bispecific antibodies, emerging treatments may be effective for treating SCLC, and further clinical trials should be carried out.

Currently, SCLC has always been treated as a single disease entity. The classification of SCLC is diverse and uncertain. More accurate and rigorous subtypes of SCLC should be determined. Moreover, biomarkers and precise treatments for different subtypes should also be elucidated.

## Data Availability

No datasets were generated or analysed during the current study.

## Abbreviations

SCLC: Small cell lung cancer
NSCLC: Non-small cell lung cancer
CNAs: Copy number alterations
TMBs: Tumor mutation burdens
NO_2_: Nitrogen dioxide
PM_10_: Particulate matter 10
PAHs: Polycyclic aromatic hydrocarbons
CRISPR: Clustered regularly interspaced short palindromic repeats
GEMMs: Genetically engineered mouse models
NE: Neuroendocrine
AT2: Alveolar type 2
SPC: Surfactant protein C
hESCs: Human embryonic stem cells
PNECs: Pulmonary NE cells
EGFR: Epidermal growth factor receptor
LUAD: Lung adenocarcinoma
TKI: Tyrosine kinase inhibitor
APOBEC: Apolipoprotein B mRNA editing enzyme catalytic polypeptide-like
CSCLC: Combined SCLC
WGS: Whole genome sequencing
HPV: Human papillomavirus
REST: RE1-silencing transcription factor
APM: Antigen processing and presentation machinery
PI3K: Phosphatidylinositol 3 kinase
TKO: Triple-knockout
H3K4: Histone H3 and lysine 4
CREBBP: CREB binding protein
EP300: E1A binding protein p300
HAT: Histone acetyltransferase
SVV-001: Seneca valley virus
TFs: Transcriptional factors
NEUROD1: Neurogenic differentiation factor 1
INSM1: Insulinoma-associated protein 1
YAP1: Yes-associated protein 1
ASCL1: Achaete-scute homolog 1
POU2F3: POU class 2 homeobox 3
ICIs: Immune checkpoint inhibitors
NMF: Non-negative matrix factorization
TAMs: Tumor-associated macrophages
DLL3: Delta-like ligand-3 (δ-like ligand 3)
RTKs: Receptor tyrosine kinases
CCSP: Clara cell secretory protein,
scRNA-seq: Single-cell RNA sequencing
AURKA/B: Aurora kinase A/B
ChIP-seq: Chromatin immunoprecipitation sequencing
Rova-T: Rovalpituzumab teserine
HDACs: Histone deacetylases
LSD1: Lysine-specific demethylase 1
SLFN11: Schlafen family member 11
PARP: Poly (ADP-ribose) polymerase
CEACAM5: Carcinoembryonic antigen-related cell adhesion molecule 5
IMPDH1 and IMPDH2: Inosine monophosphate dehydrogenase-1 and -2
CHK1: Checkpoint kinase 1
SSTR2: Somatostatin receptor 2
IGF1R: Insulin-like growth factor 1 receptor
mSWI/SNF: Mammalian switch/sucrose non-fermentable
BTK: Bruton’s tyrosine kinase
ATR: Ataxia telangiectasia and Rad3-related protein
TOP1: Topoisomerase I
PDX: Patient-derived tumor xenograft
CDX: Cell-line derived xenograft
OS: Overall survival
LS-SCLC: Limited-stage SCLC
PCI: Prophylactic cranial irradiation
NCCN: National comprehensive cancer network
TOP2: Topoisomerase II
FDA: Food and drug administration
CRT: Chemoradiotherapy
CCRT: Concurrent CRT
PFS: Progression-free survival
ES-SCLC: Extensive-stage SCLC
CSCO: Chinese society of clinical oncology
NMPA: National medical products administration
TME: Tumor microenvironment
SIRPα: Signal-regulatory protein alpha
B7-H3: B7 homolog 3 protein
ORR: Objective response rate
CR: Complete response
PR: Partial response
NKG2A: Natural killer group 2A
DC: Dendritic cell
CIK: Cytokine-induced killer
TRT: Thoracic radiation therapy
WBRT: Whole brain radiation therapy
DDR: DNA damage repair
AEs: Adverse events
TMZ: Temozolomide
H3K27me3: Methylating lysine at position 27 on histone H3
ADC: Antibody–drug conjugate
CDK: Cyclin-dependent kinase
CTC: Circulating tumor cell
CIM: Chemotherapy-induced myelosuppression
HSPCs: Hematopoietic stem and progenitor cells
EZH2: Enhancer of Zeste Homolog 2
PRC2: Polycomb repressive complex 2
H3K27me3: Methylating lysine at position 27 on histone H3
BTLA: B- and T-lymphocyte attenuator
HVEM: Herpesvirus entry mediator
